# ﻿Ribbon worms (phylum Nemertea) from Bodega Bay, California: A largely undescribed diversity

**DOI:** 10.3897/zookeys.1204.117869

**Published:** 2024-06-04

**Authors:** Christina I. Ellison, Madeline R. Frey, Eric Sanford, Svetlana Maslakova

**Affiliations:** 1 Department of Biology, Oregon Institute of Marine Biology, University of Oregon, Charleston, OR, USA University of Oregon Charleston United States of America; 2 Bodega Marine Laboratory, Bodega Bay, CA 94923, USA Bodega Marine Laboratory Bodega Bay United States of America; 3 Department of Evolution and Ecology, University of California, Davis, CA 95616, USA University of California Davis United States of America

**Keywords:** Biodiversity, cryptic species, Hoplonemertea, invertebrate, marine, Palaeonemertea, Pilidiophora

## Abstract

The diversity of nemerteans along the Pacific coast of the United States is regarded as well characterized, but there remain many cryptic, undescribed, and “orphan” species (those known only in their larval form). Recent sampling of nemerteans in Oregon and Washington has begun to fill in these taxonomic gaps, but nemertean diversity in California has received relatively little attention over the past 60 years. During the summers of 2019 and 2020, nemertean specimens were collected from 20 locations in the Bodega Bay region of northern California, USA, including rocky intertidal shores, sandy beaches, mudflats, and other habitats. Based on morphological assessment and DNA sequence analysis (partial Cytochrome Oxidase I and 16S rRNA genes), our surveys identified 34 nemertean species. Only 13 of these (38%) can be confidently assigned to described species. Another 11 represent species that are new to science, including members of the genera *Riserius*, *Nipponnemertes*, *Poseidonemertes*, *Zygonemertes*, *Nemertellina*, *Oerstedia*, and three species of uncertain affiliation. The remaining ten species include undescribed or cryptic species of uncertain status that have been found previously along the Pacific Coast of North America. Our surveys also document extensions of known geographic ranges for multiple species, including the first records in California of *Antarctonemertesphyllospadicola*, *Cephalothrixhermaphroditica*, and *Maculauraoregonensis*. This is the first report of the genus *Nemertellina* in the northeast Pacific and *Riserius* in California. Overall, our findings highlight how much remains to be learned about the diversity and distribution of nemerteans in the northeast Pacific.

## ﻿Introduction

Nemerteans, or ribbon worms, are a phylum of approximately 1300 species (Gibson 1995; Kajihara et al. 2008; WoRMS Editorial Board 2023) of soft-bodied, non-segmented worms with an eversible proboscis housed within a fluid-filled rhynchocoel. They are found in all the world’s oceans, with most species known from benthic marine habitats, although some have adapted to pelagic, fresh-water, and terrestrial environments as well. Most nemerteans are predators that feed on annelids, crustaceans, and mollusks (McDermott and Roe 1985), some throughout their life cycle, i.e., even in the plankton as larvae (Maslakova and Hiebert 2014; von Dassow et al. 2022; Maslakova et al. 2024). Some nemertean species impact commercially important crab, clam, and eel fisheries (e.g., Kuris and Wickham 1987; Bourque et al. 2001; Park et al. 2019), and some have been shown to produce compounds with biomedical potential (Kem et al. 2006; Whelan et al. 2014; Göransson et al. 2019; Verdes et al. 2022). Despite their ecological and applied importance, nemerteans remain understudied as a group, and the vast majority of the phylum’s diversity has yet to be characterized (e.g., Mahon et al. 2010; Chernyshev and Lutaenko 2011; Kajihara 2017; Maslakova et al. 2022).

Nemerteans are challenging to identify given their relatively small number of external features, many of which do not fall into discrete character states (e.g., color and shape of body), and the fact that many species have been described and are known only from histological study of formalin-preserved material. The phylum is known to possess large numbers of cryptic species, that is, morphologically indistinguishable, but nevertheless distinct, species (e.g., Chen et al. 2010; Leasi and Norenburg 2014; Hao et al. 2015; Hiebert and Maslakova 2015a). Given that morphological approaches often fail to distinguish between look-alikes, DNA-based approaches are increasingly used to identify and delimit species, resolve phylogenies, and infer biogeographical patterns (e.g., Chernyshev et al. 2021a; Mendes et al. 2021; Kajihara et al. 2022a, 2022b).

The nemertean fauna of the Cold Temperate Northern Pacific province (as per Spalding et al. 2007) is among the best-characterized in the world, as it has been studied by several nemertinologists during the course of the past century and a half (e.g., Stimpson 1857; Griffin 1898; Coe 1901, 1904, 1905, 1940; Corrêa 1964). However, new nemertean species are routinely discovered by researchers working in this region as molecular techniques are applied, and as new locations, life stages, habitats and depths are sampled and living specimens studied (e.g., Maslakova and von Dassow 2012; Hiebert et al. 2013; Hao et al. 2015; Hiebert and Maslakova 2015a, 2015b; Hunt and Maslakova 2017; Chernyshev et al. 2021b). Roe et al. (2007) report 65 intertidal nemertean species for the Oregonian Biogeographic Province (Oregon to Central California). However, recent sampling and DNA-barcoding of adults and planktonic larvae increased the known number of nemertean species in Oregon alone to more than a hundred (Hiebert 2016; Maslakova et al. 2022). In contrast, the diversity of nemerteans in California has received relatively little attention during the past 60 years, and molecular methods have not been applied broadly to the nemertean fauna of California.

The Bodega Bay region in northern California supports biodiverse ecosystems including rocky shores, kelp forests, sandy beaches, seagrass beds, and mudflats. The diversity of nemerteans in the Bodega Bay region has received some attention historically. A student report by Tamura (1957) identified 12 nemertean species in this region. Corrêa (1964) surveyed the southern end of Bodega Bay, including Dillon Beach, Tomales Point, and Tomales Bay, and identified 18 nemertean species. A pair of surveys conducted of Bodega Harbor (Standing et al. 1975) and the open coast of Bodega Marine Reserve (Ristau et al. 1978) identified seven nemertean species. Collectively, these previous studies identified a total of 21 different species of nemerteans in this region. The goal of this study was to extend the geographic scope of recent investigations of nemertean diversity in the northeast Pacific (Hiebert 2016; Maslakova et al. 2022) to include northern California. In particular, we collected nemerteans from a variety of habitats in the Bodega Bay region and used DNA barcoding to examine this fauna for the first time.

## ﻿Materials and methods

### ﻿Specimen collection and preservation

**Figure 1.**
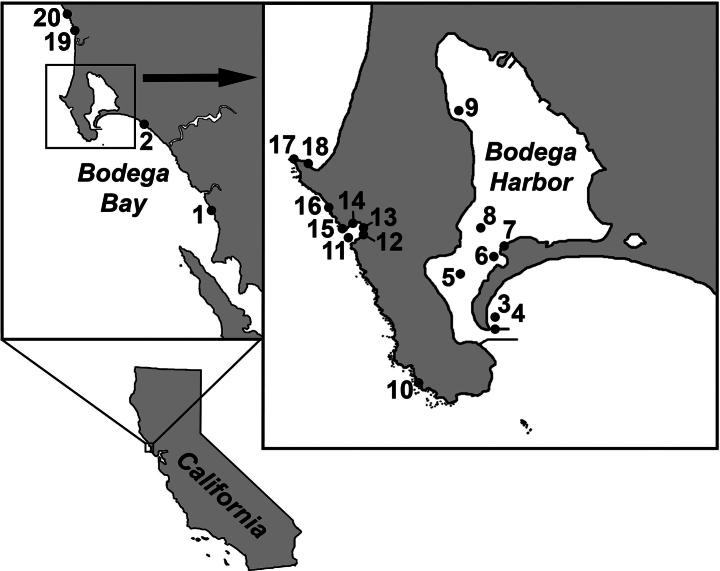
Map of collection stations (1–20) within Bodega Bay, California, USA.

During the summers of 2019 and 2020, we collected samples from 20 locations around Bodega Bay, California (Fig. 1, Tables 1, 2).

**Table 1.** Nemertean collection locations in the Bodega Bay region. Locations #8 and #11–#18 are within the Bodega Marine Reserve.

Station	GPS Coordinates	Site Description
1	38.2573, -122.9713	Dillon Beach: wave-exposed, intertidal habitat with boulders and rocky outcrops
2	38.3055, -123.0171	Pinnacle Gulch: wave-exposed, intertidal habitat with boulders and rocky outcrops
3	38.3064, -123.0514	Doran Beach: subtidal, soft sediment habitat
4	38.3059, -123.0524	Bodega Harbor North Jetty: intertidal jetty with boulders
5	38.3106, -123.0556	Bodega Harbor: intertidal flats with soft sediments
6	38.3131, -123.0514	Bodega Harbor, near Coast Guard dock: intertidal breakwater with boulders
7	38.3141, -123.0515	Bodega Harbor, near Coast Guard dock: intertidal breakwater with boulders
8	38.3135, -123.0543	Bodega Harbor, Gaffney Point: intertidal flats with soft sediments
9	38.3289, -123.0573	Bodega Harbor, Spud Point Marina: subtidal fouling community on floating docks
10	38.3002, -123.0617	Bodega Head, south end: wave-exposed, rocky intertidal habitat with shaded caves
11	38.3150, -123.0712	Bodega Head, Horseshoe Cove: subtidal holdfasts of bull kelp (*Nereocystisluetkeana*)
12	38.3159, -123.0693	Bodega Head, south side of Horseshoe Cove: rocky intertidal benches, among the roots of surfgrass (*Phyllospadix* sp.)
13	38.3162, -123.0691	Bodega Head, Horseshoe Cove Beach: intertidal sandy beach with coarse sediments
14	38.3168, -123.0709	Bodega Head, north side of Horseshoe Cove: rocky intertidal benches, among the roots of surfgrass (*Phyllospadix* sp.)
15	38.3161, -123.0719	Bodega Head, Horseshoe Cove Point: wave-exposed rocky intertidal benches at northern edge of the cove
16	38.3185, -123.0740	Bodega Head: wave-exposed rocky intertidal benches
17	38.3235, -123.0785	Bodega Head, Mussel Point: wave-exposed rocky intertidal benches
18	38.3231, -123.0766	Bodega Head: rocky intertidal benches south of Salmon Creek Beach
19	38.3631, -123.0709	Coleman Beach: wave-exposed, intertidal habitat with boulders and rocky outcrops
20	38.3747, -123.0789	Schoolhouse Beach: wave-exposed, intertidal habitat with boulders and rocky outcrops

**Table 2.** Species identity, collection location, and accession numbers of nemertean specimens from Bodega Bay, CA. “Station” refers to collection location (Fig. 1, Table 1), USNM (United States National Museum) numbers refers to vouchers deposited at the Smithsonian Institution’s National Museum of Natural History.

Specimen ID	OTU (ASAP subset)	Species ID	Station	BOLD process ID	GenBank accession number	USNM voucher
**B01**	**BOBA001**	*Maculauraoregonensis* Hiebert & Maslakova, 2015a	6	NONEP001-21	COI: OQ075685 16S: OQ075747	USNM 1673940
**B02**	**BOBA009**	*Paranemertes* sp. BOBA009	6	NONEP002-21	COI: OQ075698 16S: OQ075759	USNM 1673941
**B03**	**BOBA011**	*Nemertellina* sp. BOBA011	6	NONEP003-21	COI: OQ075690 16S: OQ075753	N/A
**B04**	**BOBA029**	*Tetrastemma* sp. BOBA029	6	NONEP004-21	COI: OQ075707	N/A
**B05**	**BOBA012**	*Zygonemertes* sp. BOBA012	6	NONEP005-21	COI: OQ075710 16S: OQ075767	N/A
**B06**	**BOBA012**	*Zygonemertes* sp. BOBA012	6	NONEP006-21	COI: OQ075709	N/A
**B07**	**BOBA014**	*Zygonemertes* sp. BOBA014	6	NONEP007-21	COI: OQ075721 16S: OQ075778	N/A
**B08**	**BOBA014**	*Zygonemertes* sp. BOBA014	6	NONEP008-21	COI: OQ075717 16S: OQ075774	N/A
**B09**	**BOBA012**	*Zygonemertes* sp. BOBA012	6	NONEP009-21	16S: OQ075773	N/A
**B10**	**BOBA031**	*Tubulanussexlineatus* (Griffin, 1898)	10	NONEP010-21	COI: OQ075708 16S: OQ075766	USNM 1673942
**B11**	**BOBA007**	*Riserius* sp. BOBA007	13	NONEP011-21	COI: OQ075703 16S: OQ075764	USNM 1673943
**B12**	**BOBA025**	*Cephalothrixsimula* (Iwata, 1952)	9	NONEP012-21	COI: OQ075671 16S: OQ075732	USNM 1673944
**B13**	**BOBA026**	*Cephalothrixhermaphroditica* (Gibson, Sánchez & Méndez, 1990)	15	NONEP013-21	COI: OQ075668 16S: OQ075729	USNM 1673945
**B14**	**BOBA010**	*Poseidonemertes* sp. BOBA010	3	NONEP014-21	COI: OQ075700 16S: OQ075761	USNM 1673946
**B15**	**BOBA016**	Eumonostilifera sp. BOBA016	9	NONEP015-21	COI: OQ075687 16S: OQ075750	USNM 1673947
**B16**	**BOBA027**	*Emplectonemaviride* Stimpson, 1857	1	NONEP016-21	COI: OQ075674 16S: OQ075735	USNM 1673948
**B18**	**BOBA021**	*Tetrastemmabilineatum* Coe, 1904	1	NONEP017-21	COI: OQ075705	USNM 1673949
**B19**	**BOBA006**	*Maculauracerebrosa* Hiebert & Maslakova, 2015	16	NONEP018-21	COI: OQ075682 16S: OQ075743	USNM 1673950
**B20**	**BOBA017**	*Amphiporus* sp. BOBA017	16	NONEP019-21	COI: OQ075662 16S: OQ075723	USNM 1673951
**B21**	**BOBA011**	*Nemertellina* sp. BOBA011	9	NONEP020-21	COI: OQ075691 16S: OQ075754	N/A
**BON01**	**BOBA019**	*Tetrastemmanigrifrons* Coe, 1904	17	NONEP021-21	COI: OQ075706	USNM 1673952
**BON02**	**BOBA002**	*Micruraverrilli*	18	NONEP022-21	COI: OQ075686 16S: OQ075748	USNM 1673953
**BON03**	**BOBA003**	*Kulikovia* sp. BOBA003	10	NONEP023-21	COI: OQ075675 16S: OQ075736	USNM 1673954
**BON04**	**BOBA026**	*Cephalothrixhermaphroditica* (Gibson, Sánchez & Méndez, 1990)	10	NONEP024-21	COI: OQ075670 16S: OQ075731	USNM 1673955
**BON06**	**BOBA005**	*Lineusflavescens* Coe, 1904	6	NONEP025-21	COI: OQ075679 16S: OQ075740	USNM 1673956
**BON10**	**BOBA001**	*Maculauraoregonensis* Hiebert & Maslakova, 2015a	8	NONEP026-21	16S: OQ075746	USNM 1673957
**BON11**	**BOBA005**	*Lineusflavescens* Coe, 1904	8	NONEP027-21	COI: OQ075681 16S: OQ075742	USNM 1673958
**BON13**	**BOBA028**	*Nipponnemertes* sp. BOBA028	7	NONEP028-21	COI: OQ075693 16S: OQ075756	USNM 1673959
**BON16**	**BOBA018**	*Amphiporus* sp. BOBA018	19	NONEP029-21	COI: OQ075660 16S: OQ075722	USNM 1673960
**BON24**	**BOBA032**	*Carinomellalactea* Coe, 1905	8	NONEP030-21	COI: OQ075667 16S: OQ075728	USNM 1673961
**BON27**	**BOBA013**	*Zygonemertes* sp. BOBA013	7	NONEP031-21	COI: OQ075716 16S: OQ075772	N/A
**BON32**	**BOBA022**	*Oerstedia* sp. BOBA022	4	NONEP032-21	COI: OQ075694 16S: OQ075757	N/A
**BON33**	**BOBA022**	*Oerstedia* sp. BOBA022	4	NONEP033-21	COI: OQ075695 16S: OQ075758	N/A
**BON35**	**BOBA033**	*Poseidonemertes* sp. BOBA033	8	NONEP034-21	COI: OQ075699 16S: OQ075760	USNM 1673962
**BON36**	**BOBA024**	*Amphiporus* sp. BOBA024	18	NONEP035-21	COI: OQ075658	USNM 1673963
**BON38**	**BOBA026**	*Cephalothrixhermaphroditica* (Gibson, Sánchez & Méndez, 1990)	18	NONEP036-21	COI: OQ075669 16S: OQ075730	USNM 1673964
**BON40**	**BOBA005**	*Lineusflavescens* Coe, 1904	2	NONEP037-21	COI: OQ075680 16S: OQ075741	USNM 1673965
**BON41**	**BOBA034**	*Micrurawilsoni* (Coe, 1904)	7	NONEP038-21	16S: OQ075749	USNM 1673966
**BON44**	**BOBA018**	*Amphiporus* sp. BOBA018	18	NONEP039-21	COI: OQ075663	USNM 1673967
**BON47**	**BOBA017**	*Amphiporus* sp. BOBA017	18	NONEP040-21	COI: OQ075661	USNM 1673968
**BON50**	**BOBA005**	*Lineusflavescens* Coe, 1904	2	NONEP041-21	COI: OQ075676 16S: OQ075737	USNM 1673969
**BON51**	**BOBA025**	*Cephalothrixsimula* (Iwata, 1952)	9	NONEP042-21	COI: OQ075672 16S: OQ075733	USNM 1673970
**BON59**	**BOBA004**	Siphonenteron gen. sp. BOBA004	15	NONEP043-21	COI: OQ075704 16S: OQ075765	USNM 1673971
**BON60**	**BOBA005**	*Lineusflavescens* Coe, 1904	15	NONEP044-21	COI: OQ075677 16S: OQ075738	USNM 1673972
**BON61**	**BOBA024**	*Amphiporus* sp. BOBA024	14	NONEP045-21	COI: OQ075659	USNM 1673973
**BON62**	**BOBA015**	*Zygonemertes* sp. BOBA015	14	NONEP046-21	COI: OQ075715	USNM 1673974
**BON63**	**BOBA015**	*Zygonemertes* sp. BOBA015	14	NONEP047-21	COI: OQ075714 16S: OQ075771	N/A
**BON64**	**BOBA007**	*Riserius* sp. BOBA007	13	NONEP048-21	COI: OQ075701 16S: OQ075762	USNM 1673975
**BON65**	**BOBA007**	*Riserius* sp. BOBA007	13	NONEP049-21	COI: OQ075702 16S: OQ075763	USNM 1673976
**BON67**	**BOBA023**	*Antarctonemertesphyllospadicola* (Stricker, 1985)	14	NONEP050-21	COI: OQ075664 16S: OQ075724	USNM 1673977
**BON68**	**BOBA023**	*Antarctonemertesphyllospadicola* (Stricker, 1985)	14	NONEP051-21	COI: OQ075665 16S: OQ075725	USNM 1673978
**BON69**	**BOBA011**	*Nemertellina* sp. BOBA011	14	NONEP052-21	COI: OQ075689 16S: OQ075752	N/A
**BON70**	**BOBA008**	*Cerebratulus* sp. BOBA008	5	NONEP053-21	COI: OQ075673 16S: OQ075734	USNM 1673979
**BON75**	**BOBA020**	*Tetrastemma* sp. BOBA020	13	NONEP054-21	COI: OQ075688 16S: OQ075751	N/A
**BON76**	**BOBA013**	*Zygonemertes* sp. BOBA013	10	NONEP055-21	COI: OQ075718 16S: OQ075775	USNM 1673986
**BON77**	**BOBA030**	*Ototyphlonemertes* sp. BOBA030	13	NONEP056-21	COI: OQ075696	N/A
**BON78**	**BOBA030**	*Ototyphlonemertes* sp. BOBA030	13	NONEP057-21	COI: OQ075697	N/A
**BON80**	**BOBA013**	*Zygonemertes* sp. BOBA013	10	NONEP058-21	COI: OQ075719 16S: OQ075776	USNM 1673980
**BON81**	**BOBA015**	*Zygonemertes* sp. BOBA015	10	NONEP059-21	COI: OQ075720 16S: OQ075777	USNM 1673981
**BON83**	**BOBA006**	*Maculauracerebrosa* Hiebert & Maslakova, 2015a	12	NONEP060-21	COI: OQ075684 16S: OQ075745	USNM 1673982
**BON85**	**BOBA006**	*Maculauracerebrosa* Hiebert & Maslakova, 2015a	12	NONEP061-21	COI: OQ075683 16S: OQ075744	USNM 1673983
**BON86**	**BOBA005**	*Lineusflavescens* Coe, 1904	10	NONEP062-21	COI: OQ075678 16S: OQ075739	USNM 1673984
**BON87**	**BOBA013**	*Zygonemertes* sp. BOBA013	11	NONEP063-21	COI: OQ075711 16S: OQ075768	N/A
**BON88**	**BOBA014**	*Zygonemertes* sp. BOBA014	6	NONEP064-21	COI: OQ075712 16S: OQ075769	USNM 1673985
**BON91**	**BOBA014**	*Zygonemertes* sp. BOBA014	6	NONEP065-21	COI: OQ075713 16S: OQ075770	N/A
**BON93**	**BOBA032**	*Carinomellalactea* Coe, 1905	8	NONEP066-21	16S: OQ075726	USNM 1673987
**BON94**	**BOBA032**	*Carinomellalactea* Coe, 1905	8	NONEP067-21	COI: OQ075666 16S: OQ075727	USNM 1673988
**BON95**	**BOBA028**	*Nipponnemertes* sp. BOBA028	7	NONEP068-21	COI: OQ075692 16S: OQ075755	USNM 1673989

We selected the locations to encompass a variety of habitat types, including rocky substrate and soft sediments in both wave-exposed/outer coast and wave-protected, estuarine/harbor environments. Most of our collections were from the intertidal zone, but some subtidal collections were made as well. In the field, we haphazardly searched for visible nemerteans and additionally collected complex habitat material to extract small worms in the laboratory. Complex material (e.g., sedentary/colonial animals, algal holdfasts, surfgrass roots, sand, mud, etc.) was collected in the field and placed into sealed plastic bags. In the laboratory, we transferred these materials into clear aquaria, and covered the material with seawater. These samples were left for several days and checked regularly to remove any nemerteans observed crawling on the walls or water’s surface. After several days, we broke apart and sorted through complex habitat material to remove hidden worms. Colonial ascidians and other invertebrates from samples of benthic communities tended to deteriorate rapidly in the laboratory and caused any nemertean specimens to become unusable, so these materials were sorted soon after collection.

Photographs and videos of worms were taken using a Leica MC170HD digital camera mounted to a Leica M125 dissecting microscope or a Leica DM1000 compound microscope, with accompanying software (Leica Application Suite v. 4.4). For hoplonemerteans, the stylet and basis of living specimens were photographed, when possible, using a compound microscope. We made initial species identifications using available morphological keys and geographically relevant inventories of Nemertea (Coe 1905; Roe et al. 2007; Hiebert 2016). Tissue samples were then preserved in 95% ethanol for DNA extraction, and some specimens were preserved in 4% formalin as morphological vouchers. Specimens collected in 2019 were identified with numbers preceded by the letter “B” and those collected in 2020 were given identifiers beginning with the label “BON.” Morphological and tissue vouchers have been deposited in the National Museum of Natural History, Smithsonian Institution, Washington, D.C. (Table 2, see also BOLD dataset https://dx.doi.org/10.5883/DS-NEMBBCA).

### ﻿DNA extraction, PCR, sequence analysis, and species delimitation

We extracted DNA from 76 individuals using DNEasy Blood and Tissue Kit (Qiagen) following the manufacturer’s protocol. We attempted to PCR–amplify portions of two mitochondrial genes, cytochrome c oxidase subunit I (COI) and 16S rRNA, from each individual, using universal and nemertean–specific primers (Table 3).

**Table 3.** PCR primers used in this study.

Locus	Primer name	Primer sequence	Reference
COI	LCO1490 HCO2198	5’ GGTCAACAAATCATAAAGATATTGG 5’ TAAACTTCAGGGTGACCAAAAAATCA	Folmer et al. 1994
COI	COI LF	5’ TTTCAACAAATCATAAAGATAT 5’ GAGAAATAATACCAAAACCAGG	Cherneva et al. 2023
	COI DR		
16S	16SARL 16SBRH	5’ CGCCTGTTTATCAAAAACAT 5’ CCGGTCTGAACTCAGATCACGT	Palumbi et al. 1991
16S	16S AF 16S KR	5’ TCGTCTGTTTATCAAAAACATAGY 5’ AATAGATAGAAACCAACCTGGC	Cherneva et al. 2023

Each PCR was performed in a 20 μl volume, with 1 unit per reaction of Go Taq Polymerase (Promega) with supplied buffer, 200 μM dNTPs, and 500 nM of each primer. We used the following thermocycle profile: 95 °C 2 min; 34 cycles of: 95 °C 40 s, 45 °C (COI) 48–50 °C (16S) 40 s, and 72 °C 1 min; followed by final extension for 2 min (72 °C). Some DNA extracts required dilution (~ 1:20) for PCR success. PCR products were assessed with gel electrophoresis, purified with Wizard SV Gel and PCR Clean Up Kit (Promega), and sequenced at Sequetech (Mountain View, CA) in both directions using PCR primers. We used Geneious Prime for sequence analysis. Sequences with initial HQ < 50% were discarded. Each sequence was manually trimmed to eliminate primers and low–quality end regions. Forward and reverse strands were aligned, proofread against each other using quality PHRED scores and chromatograms, and contigs used to generate consensus sequences. Nucleotides with combined PHRED score of less than 20 in consensus sequences were trimmed off or converted to “N”. We translated each COI nucleotide sequence into amino acids using the Invertebrate Mitochondrial translation table and checked for the presence of stop codons.

Consensus sequences were checked against the NCBI database (GenBank) using nucleotide BLAST to screen for contamination and to aid with specimen identification. A 4% p-distance divergence was previously identified as appropriate for species delineation in large scale COI-barcoding studies of nemerteans (e.g., Kvist et al. 2014; Maslakova et al. 2022), and thus was used here as a criterion for selection of conspecific reference sequences. When available, conspecific sequences from GenBank were added to the alignment. Reference sequences from closely related species were added where no conspecific reference sequences were available (Suppl. material 1). All newly generated sequences have been deposited in BOLD and GenBank (Table 2, see also BOLD dataset https://dx.doi.org/10.5883/DS-NEMBBCA). We aligned sequences using the MAFFT plug-in within Geneious, using default parameters, visually inspected alignments for gaps and irregularities, then used them to construct unrooted neighbor–joining trees (Tamura Nei substitution model). Final COI alignment was trimmed to 658 bp and contained a total of 107 sequences (including 64 generated in this study), and 16S alignment was 568 bp long, and contained 93 sequences (57 generated in this study). We used ASAP (Assembling Species by Automatic Partitioning) analysis (Puillandre et al. 2021) of the COI data to partition the dataset into Operational Taxonomic Units (OTUs), putative species. The 16S rRNA sequences were used to verify species identity, where we lacked COI sequence data, but were not used for species delineation, because they are more conservative and can fail to differentiate between closely related species. Below we refer to reference sequences by either GenBank accession number or BOLD Process ID, where accession numbers are not available.

## ﻿Results

**Figure 2.**
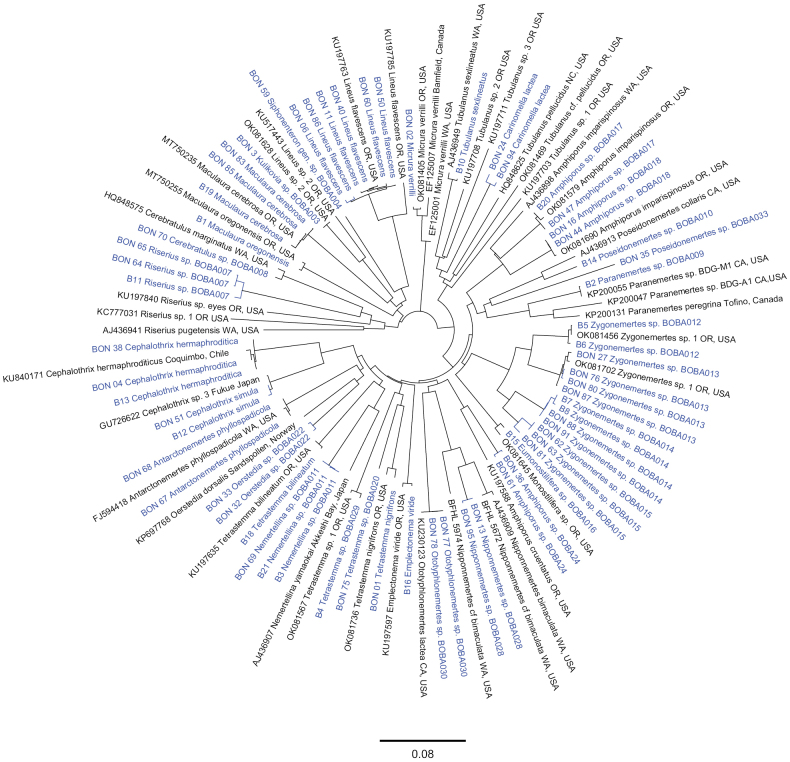
Neighbor-joining tree of COI sequences. Sequences generated in this study are shown in blue. BOBA numbers reflect subsets (OTUs) from the ASAP analysis.

**Figure 3.**
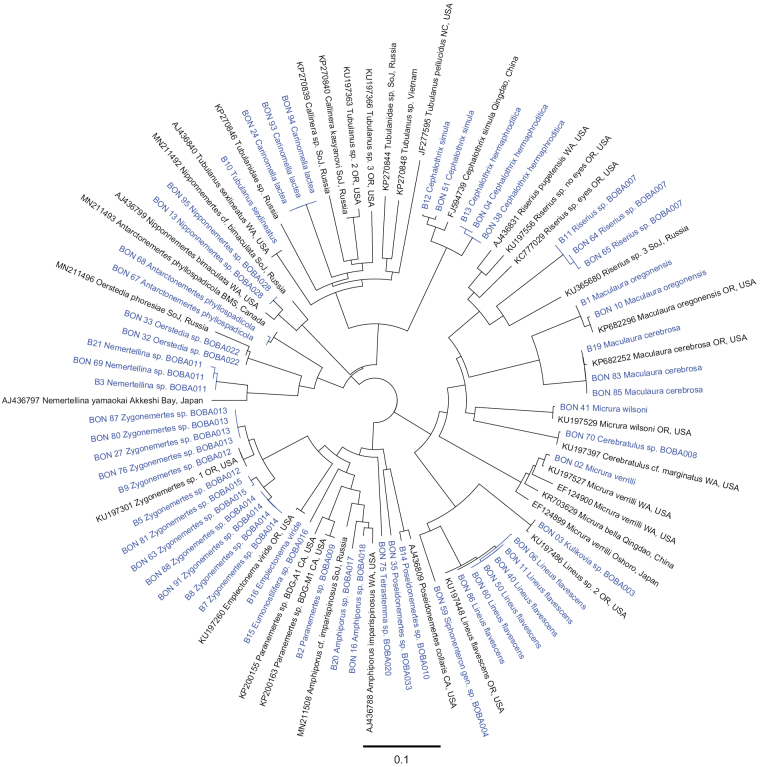
Neighbor-joining tree of 16S sequences. Sequences generated in this study are shown in blue. BOBA numbers reflect subsets (OTUs) from ASAP analysis on COI data.

Of the 76 specimens from which DNA was extracted, we were able to successfully obtain sequences from 68; a total of 64 COI sequences and 57 16S sequences (see Table 2, BOLD dataset https://dx.doi.org/10.5883/DS-NEMBBCA). Results of the ASAP analysis of the COI data suggested the presence of a barcoding gap (i.e., the separation between the maximum intraspecific and the minimum interspecific variation) between 3–5%, similar to previous large-scale DNA-barcoding studies of nemerteans (e.g., Kvist et al. 2014; Sundberg et al. 2016; Maslakova et al. 2022). Using 0.038 simple p-distance as the threshold, ASAP partitioned the dataset into 52 OTUs, 33 of which contained all our samples (Table 4). BOLD species delineation algorithm partitioned our sequences into 34 barcode index numbers (BINs, Table 4), splitting *Zygonemertes* sp. BOBA015 into two separate BINs. Of the 34 BINs, ten are unique, meaning they lacked representation in BOLD (likely not previously sequenced). We were not able to obtain a COI sequence from specimen BON41, but its identity was determined morphologically as *Micrurawilsoni* (Coe, 1904) and was confirmed by the 16S rRNA sequence data. There is no evidence of cryptic species of *M.wilsoni* in the region thus far. Hence, we report a total of 34 putative species of nemerteans from Bodega Bay, CA (Table 4). COI sequences were obtained for 33/34 species, and 16S sequences for 28/34 species. Neighbor–Joining trees of the COI and 16S rRNA sequence alignments are shown on Figs 2, 3, respectively.

**Table 4.** Nemertean species identified from Bodega Bay, California. The term “unresolved” refers to cryptic species of uncertain taxonomic status.

Class	Species	Status	OTU code (this study)	BOLD Barcode Identification Number (BIN)	Maslakova et al. (2022) OTU
Palaeonemertea	*Cephalothrixhermaphroditica* (Gibson, Sánchez & Méndez, 1990)	described	BOBA026	BOLD:ADM3467	-
Palaeonemertea	*Cephalothrixsimula* (Iwata, 1952)	described	BOBA025	BOLD:AAM5519	-
Palaeonemertea	*Carinomellalactea* Coe, 1905	described	BOBA032	BOLD:AEJ8707	-
Palaeonemertea	*Tubulanussexlineatus* (Griffin, 1898)	described	BOBA031	BOLD:ADM0945	OTU 83
Pilidiophora	*Cerebratulus* sp. BOBA008	previously reported, undescribed	BOBA008	BOLD:AAE9633	OTU 62
Pilidiophora	*Kulikovia* sp. BOBA003	previously reported, undescribed	BOBA003	BOLD:ADX1401	OTU 49
Pilidiophora	*Maculauracerebrosa* Hiebert & Maslakova, 2015a	described	BOBA006	BOLD:AAP1201	OTU 54
Pilidiophora	*Maculauraoregonensis* Hiebert & Maslakova, 2015a	described	BOBA001	BOLD:ADM2641	OTU 61
Pilidiophora	*Riserius* sp. BOBA007	new to science	BOBA007	BOLD:AEJ1230	-
Pilidiophora	*Lineusflavescens* Coe, 1904	described	BOBA005	BOLD:ADS0049	OTU 45
Pilidiophora	*Micruraverrilli* Coe, 1901	described	BOBA002	BOLD:ADW4746	OTU 65
Pilidiophora	*Micrurawilsoni* (Coe, 1904)	described	BOBA034	BOLD:ADW9830	OTU 90
Pilidiophora	Siphonenteron gen. sp. BOBA004	new to science	BOBA004	BOLD:ADR9817	-
Hoplonemertea	*Nipponnemertes* sp. BOBA028	new to science	BOBA028	BOLD:AEJ7531	-
Hoplonemertea	*Amphiporus* sp. BOBA024	new to science	BOBA024	BOLD:AEI5687	-
Hoplonemertea	*Amphiporus* sp. BOBA017	previously reported, undescribed	BOBA017	BOLD:ADR7530	OTU 6
Hoplonemertea	*Amphiporus* sp. BOBA018	previously reported, undescribed	BOBA018	BOLD:AEA1922	OTU 5
Hoplonemertea	*Emplectonemaviride* Stimpson, 1857	described	BOBA027	BOLD:AAP1200	OTU 17
Hoplonemertea	*Ototyphlonemertes* sp. BOBA030	previously reported, unresolved	BOBA030	BOLD:ADM3126	-
Hoplonemertea	*Paranemertes* sp. BOBA009	previously reported, undescribed	BOBA009	BOLD:ADM0221	OTU 10
Hoplonemertea	*Poseidonemertes* sp. BOBA010	new to science	BOBA010	BOLD:AEK1697	-
Hoplonemertea	*Poseidonemertes* sp. BOBA033	new to science	BOBA033	BOLD:AEK1698	-
Hoplonemertea	*Tetrastemmanigrifrons* Coe, 1904	described	BOBA019	BOLD:ADX0572	OTU 18
Hoplonemertea	*Tetrastemma* sp. BOBA029	previously reported, undescribed	BOBA029	BOLD:ADW8618	OTU 20
Hoplonemertea	*Tetrastemma* sp. BOBA020	new to science	BOBA020	BOLD:AEJ7493	-
Hoplonemertea	*Zygonemertes* sp. BOBA012	previously reported, unresolved	BOBA012	BOLD:ADL9636	OTU 23
Hoplonemertea	*Zygonemertes* sp. BOBA013	previously reported, unresolved	BOBA013	BOLD:ADW7912	OTU 26
Hoplonemertea	*Zygonemertes* sp. BOBA014	new to science	BOBA014	BOLD:AEK0256	-
Hoplonemertea	*Zygonemertes* sp. BOBA015	new to science	BOBA015	BOLD:AEJ0120, BOLD:ADR7155	-
Hoplonemertea	Eumonostilifera sp. BOBA016	previously reported, undescribed	BOBA016	BOLD:AEJ6897	OTU 13
Hoplonemertea	*Antarctonemertesphyllospadicola* (Stricker, 1985)	described	BOBA023	BOLD:ACH3602	-
Hoplonemertea	*Nemertellina* sp. BOBA011	new to science	BOBA011	BOLD:AEJ4336	-
Hoplonemertea	*Oerstedia* sp. BOBA022	new to science	BOBA022	BOLD:AEJ2779	-
Hoplonemertea	*Tetrastemmabilineatum* Coe, 1904	described	BOBA021	BOLD:ADW8130	OTU 29

Because of the ubiquitous presence of cryptic species among nemerteans, species distributions listed below refer to reports verified by DNA sequence data, unless otherwise noted. Undescribed species, as well as species of uncertain status, were assigned temporary alphanumeric OTU codes (e.g., BOBA0XX) for tracking purposes, until their taxonomy is resolved.

### ﻿Class PALAEONEMERTEA

**Order Archinemertea Iwata, 1960**

**Family Cephalotrichidae McIntosh, 1873**

**Genus *Cephalothrix* Örsted, 1843**

A species-rich genus of mostly white, thread-like worms, which have a long pre-oral region and lack ocelli as adults. Given their general lack of distinguishing features, the species therein are difficult to differentiate morphologically (e.g., Chen et al. 2010; Leasi and Norenburg 2014; Kajihara 2019; Sagorny et al. 2019). COI sequences are available for at least ten species from Oregon and California, most of which have not been described and some are only known in their larval form (Hiebert 2016; Maslakova et al. 2022).

***Cephalothrixhermaphroditica* (Gibson, Sánchez & Méndez, 1990)**

*Procephalothrixhermaphroditicus*Gibson et al., 1990: 279, figs 1–15; Sundberg and Hylbom 1994: 358; McDermott 2001: 12; Carroll et al. 2003: 52; Sundberg et al. 2003: 281.

*Cephalothrixhermaphroditicus*: Paule et al. 2021: 5.

**BIN.**BOLD:ADM3467.

**Material examined.** B13, BON4, BON38.

**Figure 4.**
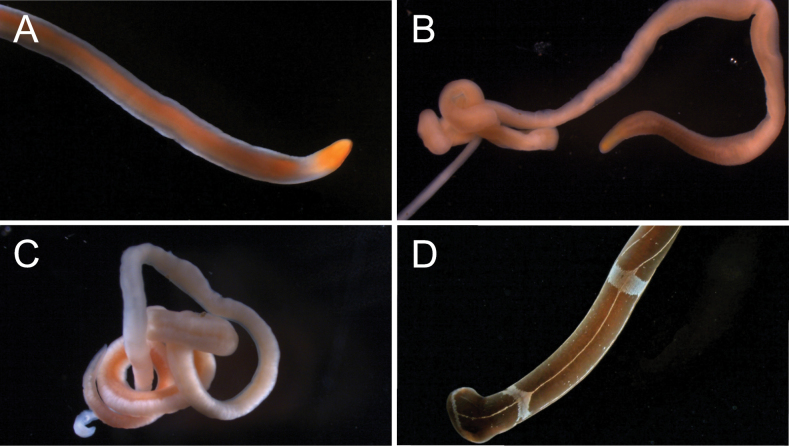
Palaeonemerteans of Bodega Bay **A***Cephalothrixhermaphroditica*, individual B13 **B***Cephalothrixsimula*, individual BON51 **C***Carinomellalactea*, individual BON24 **D***Tubulanussexlineatus*, individual B10.

**Morphology.** Filiform body, 15–51 mm long. Body color orange with translucent margins, somewhat paler ventrally, with a deeper orange anterior tip (Fig. 4A). Preoral region relatively shorter than in other *Cephalothrix* species.

**Identification.** Our specimens share high sequence similarity (99–100% COI) with specimens reported as *C.hermaphroditicus* from Chile (KU840171), France (MH681952), Spain (KM230034), and Argentina (KM230037). The type locality of the species is Cocholgue, Chile, but given the comparatively low haplotype diversity in Chile, and high haplotype diversity in France, Sagorny et al. (2019) suggested the species may have been introduced to Chile from Europe. COI barcodes for the Bodega Bay specimens are identical to the Chilean ones, consistent with the idea of a single introduction to the Pacific from the Atlantic.

**Habitat.** Collected from wave-exposed, rocky intertidal habitats, among colonial ascidians. Stations 10, 15, 18 (Fig. 1, Table 1).

**Distribution.** Bodega Bay, CA, USA (this study); Coquimbo, Chile (Sundberg et al. 2016); Camarones Bay, Argentina; Vilan Cape, Spain (Fernández-Álvarez et al., unpublished); Roscoff, France (Sagorny et al. 2019; Paule et al. 2021).

**Notes.** The only available sequences of *Cephalothrixhermaphroditica* from Chile were collected ~ 1,000 km north of its type locality. Given that biogeographical patterns among nemertean species are variable, we tentatively include the species as synonymous with the species we encountered in our surveys. This is the first report of the species in the Cold Temperate Northern Pacific, and first 16S barcode for the species. *Cephalothrixhermaphroditica* provides the only known example of hermaphrodism among the Palaeonemertea. Reproductive features were not observed by us.

***Cephalothrixsimula* (Iwata, 1952)**

*Procephalothrixsimulus* Iwata, 1952: 132.

*Cephalothrixsimula*: Kajihara et al. 2013: 987, figs 2–11 (see publication for full synonymy up through 2013).

*Cephalothrixsimula*: Chernyshev and Polyakova 2021: 586.

*Cephalothrix* sp.: Nam and Rhee 2020: 2012.

**BIN.**BOLD:AAM5519.

**Material examined.** B12, BON51.

**Morphology.** Filiform body, ~ 50 mm long, pale yellowish to orange, color brighter in the head and foregut regions, paler and somewhat translucent posteriorly (Fig. 4B).

**Identification.** Our specimens share high (99.6% COI) sequence similarity to the topogenetype of *C.simula* (GU726622), and other *C.simula* sequences, as defined by Kajihara et al. (2013). The type locality of *C.simula* is Japan, but it has since been reported from the Pacific coast of the U.S., the Atlantic, and the Mediterranean. Remarkably, and in contrast to many other reported cases of such widespread distribution among nemertean species, these reports refer to the same species, as assessed by genetic data (Chen et al. 2010; Kajihara et al. 2013; Sagorny et al. 2019). This suggests that *C.simula* has been broadly introduced to many geographic regions.

**Habitat.** Collected among fouling organisms on marina docks (station 9).

**Distribution.** Changdao, China (Chen et al. 2010, 2011); South Korea (Chen et al. 2010; Nam and Rhee 2020; Chernyshev and Polyakova 2021); Japan: Fukue, Hiroshima, Oshoro, Seto, Shimoda (Thollesson and Norenburg 2003; Chen et al. 2010; Kajihara et al. 2013); Iturup Island, Russia (Chernyshev and Polyakova 2021); Bodega Bay, CA, USA (this study); San Diego, CA, USA (Chen et al. 2010); Spain: Aramar, Blanes, Cap de Creus, Islares, San Vicente do Mar (Fernández-Álvarez and Machordom 2013; Sagorny et al. 2019); France: Concarneau, Roscoff (Sagorny et al. 2019); Italy: Giglio, Trieste (Chen et al. 2010; Sagorny et al. 2019); Netherlands: Sint Annaland, Zierikzee (Faasse and Turbeville 2015).

**Notes.** Previously reported from southern California (Chen et al. 2010), this is the first report of the species from northern California. Following the detection of *C.simula* in the Mediterranean, the species was identified as non-native (Fernández-Álvarez and Machordom 2013), likely introduced to locations outside of East Asia in the 21^st^ century via transport of larvae and juveniles in the ballast water of ships (Chernyshev 2014) or with oyster aquaculture (e.g., Ruesink et al. 2005). Apart from being one of the few known cases of species introductions amongst the nemerteans (but see Moore et al. 2001), *C.simula* has attracted interest for the high levels of neurotoxin tetrodotoxin (TTX) found in its tissues, sparking concern about potential contamination of shellfish (e.g., Kajihara et al. 2013; Turner et al. 2018).

**Order Tubulaniformes Chernyshev, 1995**

**Family Carinomellidae Chernyshev, 1995**

**Genus *Carinomella* Coe, 1905**

A monotypic genus. Morphology resembles *Tubulanus*; likely related to *Carinella*, *Parahubrechtia* and other unpigmented tubulanids (Chernyshev et al. 2021b).

***Carinomellalactea* Coe, 1905**

*Carinomellalactea* Coe, 1905: 127, pls V–XI, figs 45–61, 63–72.

**BIN.**BOLD:AEJ8707.

**Material examined.** BON24, BON93, BON94.

**Morphology.** Eyeless white worm 65–87 mm long (Fig. 4C); spatulate head demarcated from the body by width and an indistinct transverse furrow; the mouth a small slit just posterior to the furrow on the ventral side. With large, oblong, conspicuous lateral sense organs located posterior to the tubulanid ring.

**Identification.** Specimens at hand most resemble *Carinomellalactea* described by Coe (1905) from San Pedro, California and, to a lesser extent, *Tubulanuspellucidus* (Coe, 1895). The latter was described from the coast of New England, but later also reported from Monterey Bay, San Pedro, and San Diego, California by Coe (1905). In his description of the species, Coe (1905) notes that *T.pellucidus* often co–occurs with *C.lactea* in southern California, but the former is smaller in size (10–25 mm long). The species encountered here did not locate any close sequence matches within GenBank, and there are no previously published sequences of *C.lactea*. A COI sequence (OK081469) derived from a small unpigmented tubulanid reported from Oregon by Maslakova et al. (2022: OTU 89), is distinct from those of Bodega Bay specimens, and so is the only available COI sequence (HQ848625) purported to belong to an Atlantic *T.pellucidus*, from North Carolina (Andrade et al. 2012). The 16S sequences place Bodega Bay specimens within a clade of other unpigmented tubulanids including species of *Tubulanus*, *Carinella*, and *Parahubrechtia* (Fig. 3).

**Habitat.** Mudflats. Collected among polychaete tubes on intertidal mudflats, just below the surface of the sediments (station 8).

**Distribution.** Bodega Bay, CA, USA (this study). The species has been reported from the Atlantic (Corrêa 1961), but it is unlikely this report refers to the same species.

**Notes.** Species not previously sequenced.

**Family Tubulanidae sensu Chernyshev, 2022**

**Genus *Tubulanus* Renier, 1804**

***Tubulanussexlineatus* (Griffin, 1898)**

*Carinellasexlineata* Griffin, 1898: 201, fig. 15; Coe 1904: 118; 1905: 85, pl. I, figs 2, 3.

*Carinelladinema* Coe, 1901: 15, pl. I, figs 2, 3.

**BIN.**BOLD:ADM0945.

**Material examined.** B10.

**Morphology.** Slender reddish brown worm with elaborate white markings (Fig. 4D), including a series of transverse rings, six longitudinal lines (one mid-dorsal, originating from the first ring; two paired lateral and one mid-ventral originating from the second ring), and small white dots. Paler ventrally. Spatulate head well demarcated from the body by width and a pair of transverse cerebral organ furrows; with tubulanid ring and lateral sense organs. No ocelli.

**Identification.** Morphology agrees with *Tubulanussexlineatus*, a species described from Puget Sound, Washington and Alaska, and DNA sequences show high percent similarity to previously published sequences of *T.sexlineatus.* Also resembles *T.punctatus* (Takakura, 1898) from Japan, and *T.superbus* (Kölliker, 1845) from the Mediterranean. Two described *Tubulanus* species from the northeast Pacific with similar coloration have no published sequences but can be differentiated from *T.sexlineatus* by pattern: *T.cingulatus* (Coe, 1904) is deep brown, with four dorsal longitudinal white lines, and *T.capistratus* (Coe, 1901) is brown with many narrow white rings, and only three white longitudinal lines (Coe 1901; Corrêa 1964).

**Habitat.** Relatively common in the Bodega Bay region. Collected from the wave-exposed, rocky intertidal zone, among colonial ascidians (stations 2, 10, 15), and observed among surfgrass roots (stations 12, 14), kelp holdfasts (station 7), and on the underside of rocks in pale cellophane-like tubes of its own secretion (station 2, 7).

**Distribution.** Discovery Island, BC, Canada (QHAK2597-22, QHAK2649-22 in BOLD); Puget Sound, WA, USA (Thollesson and Norenburg 2003; Andrade et al. 2012; Charleston, OR, USA (Hiebert 2016; Maslakova et al. 2022); Bodega Bay, CA, USA (this study); Malibu, CA, USA (DISA603-19 in BOLD).

### ﻿Class PILIDIOPHORA Thollesson & Norenburg, 2003

**Order Heteronemertea Bürger, 1892**

**Family Lineidae**

**Genus *Cerebratulus* Renier, 1804**

*Cerebratulus* is one of three non-monophyletic mega-genera in the Class Pilidiophora, with *Lineus* and *Micrura* (e.g., see Kajihara et al. 2022b). The morphological diagnosis of the genus is based on combinations of non-unique characters (Schwartz 2009). However, since *Cerebratulusmarginatus* is the type species of the genus, and the species below is closely related (Verdes et al. 2021), it will likely retain the generic affiliation.

***Cerebratulus* sp. BOBA008**

*Cerebratulusmarginatus*: Andrade et al. 2012: 6; Thollesson and Norenburg 2003: 408; Roe et al. 2007: 224, pl. 88, figs C, D.

Cerebratuluscf.marginatus: Hiebert 2016: 48; Verdes et al. 2021: 898; Maslakova et al. 2022.

**BIN.**BOLD:AAE9633.

**Material examined.** BON70.

**Figure 5.**
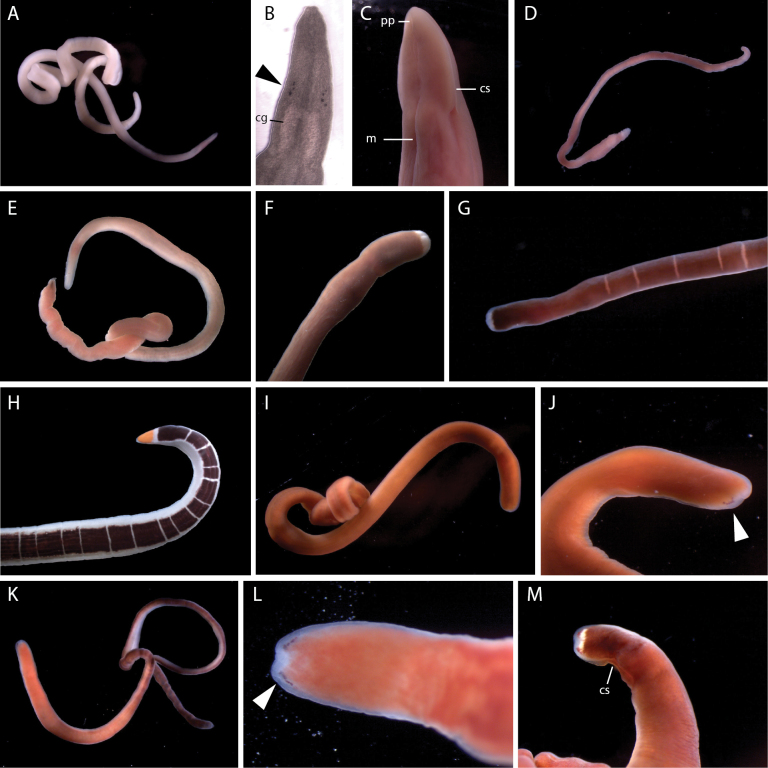
Pilidiophoran nemerteans of Bodega Bay **A, B***Riserius* sp. BOBA007, a new species, individual BON064 **B** close up of head in transmitted light showing ocelli (arrowhead) **C***Cerebratulus* sp. BOBA008, individual BON070, ventrolateral view of head **D***Maculauracerebrosa*, individual BON085 **E***Maculauraoregonensis*, individual B1 **F***Kulikovia* sp. BOBA003, anterior end of individual BON003 **G***Micrurawilsoni*, individual BON041 **H***Micruraverrilli*, individual BON002 **I, J** Siphonenteron gen. sp. BOBA004, a new species, individual BON059 **J** close up of anterior end, showing ocelli (arrowhead) **K–M***Lineusflavescens***K, L** individual BON040 **L** close up of head showing ocelli (arrowhead) **M** individual BON006, a color morph with a white anterior patch and reddish ocelli. Abbreviations: cg – cerebral ganglia, cs – cephalic slit, m – mouth, pp – proboscis pore.

**Morphology.** Large (25 cm long), dorsoventrally flattened dull reddish brown worm with lateral margins distinct both in color (pale) and shape (flattened). Spade-shaped head bordered by deep cephalic slits, with a long slit-like mouth located posterior to their endings on the ventral side (Fig. 5C). Capable of swimming. Young individuals have a pair of inconspicuous ocelli near the anterior tip of the head.

**Identification.** A common intertidal mudflat species, previously reported from Washington and Oregon as *Cerebratulusmarginatus* or Cerebratuluscf.marginatus (Thollesson and Norenburg 2003; Roe et al. 2007; Andrade et al. 2012; Hiebert 2016; Maslakova et al. 2022). The type locality of *C.marginatus* is Naples, Italy, but the closest available sequenced specimens are from Spain. Although the nominal species *C.marginatus* is reported from the eastern and western Pacific and Atlantic oceans, and the Baltic, North, and Mediterranean seas, it has been shown to constitute a large cryptic species complex with at least six lineages in Europe (Verdes et al. 2021). The seventh, NE Pacific, lineage most certainly represents an undescribed species. At least one closely related look-alike, *Cerebratulus* sp. ”spade head”, occurs in southern Oregon (Hiebert 2016; Maslakova et al. 2022: OTU 63) and another, recently re-described as *Cerebratulusorochi* Kajihara, 2020, in the Sea of Japan.

**Habitat.** Intertidal mudflats in areas with sandy sediments; often > 30 cm below the surface (station 5).

**Distribution.** Discovery Islands, BC, Canada (QHAK2554-22 in BOLD), Puget Sound, WA, USA (Thollesson and Norenburg 2003; Andrade et al. 2012; Verdes et al. 2021); Charleston, OR, USA (Hiebert 2016; Maslakova et al. 2022); Bodega Bay, CA, USA (this study).

**Genus *Kulikovia*Chernyshev et al. 2018**

***Kulikovia* sp. BOBA003**

*Lineus* sp.: Maslakova et al. 2022.

*Lineus* sp. 2: Hiebert 2016: 54, fig. 4.16.

**BIN.**BOLD:ADX1401.

**Material examined.** BON3.

**Morphology.** Body 27 cm long, reddish brown with a white anterior tip (Fig. 5F).

**Identification.** Resembles *Kulikovia* spp., and several other species in the Siphonenteron clade (see Lineidae incertae sedis below). COI sequences show that it is conspecific (99–100% similarity) with one of two sister lineages previously reported from southern Oregon in their larval form as *Lineus* sp. 2 by Hiebert (2016) and as *Lineus* sp. by Maslakova et al. (2022: OTU 49) in both larval and adult form. Phylogenetic analysis based on five genetic markers places this species within the Siphonenteron clade, more specifically within the genus *Kulikovia* (Chernyshev et al. 2018; Kajihara et al. 2022b). Two other members of the genus are reported from the northeast Pacific: *K.montgomeryi* from Kachemak, Alaska and San Juan Island, Washington (as *Cerebratulusmontgomeryi* in Schwartz 2009; Hiebert 2016), and an undescribed species reported from southern Oregon as *Lineus* sp. ‘red’ by Hiebert and Maslakova (2015b) and as *Kulikovia* sp. by Maslakova et al. (2022: OTU 59).

**Habitat.** Collected from a sandy, low intertidal pool, under stones (station 10).

**Distribution.** Charleston, OR, USA (Hiebert 2016; Maslakova et al. 2022); Bodega Bay, CA, USA (this study).

**Notes.** This is the first record of the species in California.

**Genus *Maculaura* Hiebert & Maslakova, 2015a**

The genus *Maculaura* was erected for five cryptic species occurring in NE Pacific that were previously recognized under the name *Micruraalaskensis* Coe, 1901. A phylogenetic analysis by Kajihara et al. (2022b) shows *Maculaura* as a well-supported lineid clade.

***Maculauracerebrosa* Hiebert & Maslakova, 2015a**

*Maculauracerebrosa* Hiebert & Maslakova, 2015a: 628, fig. 4G–J.

**BIN.**BOLD:AAP1201.

**Material examined.** B19, BON83, BON85.

**Morphology.** Body 15–18 mm long. Anterior tip white, with lateral cephalic slits, no ocelli, and pink cerebral ganglia visible through the body wall (Fig. 5D). One individual quite pink, with color beginning near the brain, another white throughout the foregut region, with brown coloration beginning in the intestinal area.

**Identification.** Bodega Bay individuals conform to the morphological description of *Maculaura* spp., and COI sequences exhibit 99–100% similarity to those of *M.cerebrosa* from Oregon (Hiebert and Maslakova 2015a; Maslakova et al. 2022: OTU 54).

**Habitat.** Collected from the open coast among the roots of surfgrass (*Phyllospadix* sp., station 12) and in mid-intertidal mussel beds (station 16). In southern Oregon, this species has also been found within estuaries, especially under rocks at the edges of mudflats (Hiebert and Maslakova 2015a).

**Distribution.** Wrangell, AK, USA (MOBIL9484-19 in BOLD); Bamfield, BC, Canada (OPQCS038-10 in BOLD); Charleston, OR, USA; Crescent City, CA, USA (Hiebert and Maslakova 2015a; Maslakova et al. 2022); Bodega Bay, CA, USA (this study).

***Maculauraoregonensis* Hiebert & Maslakova, 2015a**

*Maculauraoregonensis* Hiebert & Maslakova, 2015a: 630, 4L–Q.

**BIN.**BOLD:ADM2641.

**Material examined.** B1.

**Morphology.** Body ~ 18 mm long; anterior tip white, eyeless, with lateral cephalic slits, the rest of the body pink. Anterior tip rounded in extension, head not demarcated from the body. Cerebral ganglia rosy and visible through the body wall (Fig. 5E). Body somewhat transparent. Posterior end with translucent, somewhat thickened caudal cirrus.

**Identification.** Bodega Bay individuals conform to the morphological description of *Maculaura* spp., and COI sequences exhibit > 99% similarity to those of *M.oregonensis* from Oregon (Hiebert and Maslakova 2015a; Maslakova et al. 2022: OTU 61).

**Habitat.** Collected from Bodega Harbor within the holdfast of subtidal Giant Kelp (*Macrocystispyrifera*) at a depth of 3–4 m (station 6). In Oregon, individuals have also been found intertidally in sand and mud (Hiebert and Maslakova 2015a).

**Distribution.** Charleston, OR, USA (Hiebert and Maslakova 2015a; Maslakova et al. 2022); Bodega Bay, CA, USA (this study).

**Notes.** This is the first record of the species in California.

**Genus *Riserius* Norenburg, 1993**

*Riserius* is the only known genus of Pilidiophora to exhibit a mesopsammic lifestyle, i.e., living interstitially among sand grains. Its members display a curious suite of features: lack of cutis, cerebral organs opening via lateral pits rather than longitudinal slits, and a transverse cephalic furrow that encircles the body in front of the mouth. Possesses a unique sock-like pilidium recurvatum larva (Hiebert et al. 2013). A single species, *Riseriuspugetensis* Norenburg, 1993, is described from coarse marine sediments of Puget Sound, Washington. Two additional, undescribed species, *R.* sp. “eyes” and *R.* sp. “no eyes,” are known from southern Oregon, and two others, *R.* sp. 3 and *R.* sp. 4, from the Sea of Japan (Vostok Bay, Russia), all collected exclusively in larval form, but some raised in the laboratory to sexual maturity (Hiebert et al. 2013; Hiebert 2016). Similar larval forms are reported from other parts of the world, but sequence data are lacking (reviewed in Hiebert et al. 2013). Phylogenetic analysis by Kajihara et al. (2022b) suggests *Riserius* is a derived lineid that lost the lateral cephalic slits.

***Riserius* sp. BOBA007**

**BIN.**BOLD:AEJ1230.

**Material examined.** B11, BON64, BON65.

**Morphology.** Thread-like cream-colored worm, 50–100 mm long (Fig. 5A), with a long and narrow pointed head not well demarcated from the body, except by the cerebral organ pits. With four ocelli in a small cluster on each side of the head just in front of the pink tinged cerebral ganglia (Fig. 5B). Cerebral organs just posterior to the brain, opening via conspicuous lateral pits. With a V-shaped transverse furrow located posteriorly ~ 3/4 of the length from the tip of the head to the cerebral organs.

**Identification.** Morphology of our specimens agrees with that of *Riserius* spp. (Norenburg 1993; Hiebert et al. 2013), though specimens observed here are much longer than *R.pugetensis*, which is ~ 15 mm. Sequences from Bodega Bay specimens are distinct from those of the previously reported species, and 16S rRNA tree places these individuals within a monophyletic *Riserius* clade (Fig. 3). Specimens from Bodega Bay represent a new species of *Riserius*.

**Habitat.** Collected on wave-exposed, sandy beaches from among very coarse sand in the low intertidal zone at ~ -0.15 m to +0.30 m above Mean Lower Low Water (MLLW, station 13). Small numbers of individuals were also observed on other local beaches among very coarse sand (station 20).

**Distribution.** Bodega Bay, CA, USA (this study).

**Notes.** Species new to science. First record of the genus in California.

#### ﻿LINEIDAE incertae sedis

*Lineus* and *Micrura* are non-monophyletic mega-genera within the family Lineidae (e.g., see Kajihara et al. 2022b). The morphological diagnoses of these genera are based on combinations of non-unique characters (Schwartz 2009). Here we use names *Lineus and Micrura* as taxonomic artifacts, and not to imply shared common ancestry.

***Lineusflavescens* Coe, 1904**

*Lineusflavescens* Coe, 1904: 184, pl. XVII, figs 3, 4.

**BIN.**BOLD:ADS0049.

**Material examined.** BON6, BON11, BON40, BON50, BON60, BON86.

**Morphology.** Body 8–52 mm long, with significant variation in color: pale (semi-transparent), tan, reddish brown, and rosy-orange varieties observed (Fig. 5K), with lighter coloration ventrally, and smaller individuals appearing paler in color. Head not demarcated from the body, with pale margins, and 3–7 red, purple, or black ocelli arranged closely in a single row on each side of the anterior tip along the head margin (Fig. 5L, M). In some individuals, there is an irregular transverse band of white pigment granules just posterior to and between the two rows of ocelli (Fig. 5M). Cerebral ganglia are rosy, but this is faint in some individuals. Mouth just posterior to the end of the lateral cephalic slits on the ventral side. Body tapers posteriorly.

**Identification.** The six individuals included here match closely (99–100% similarity, COI) to *Lineusflavescens* reported from southern Oregon (Hiebert 2016; Maslakova et al. 2022: OTU 45) and agree with Coe’s description of the species. A closely related species (5–6% divergence, COI) is reported from Dutch Harbor, Alaska (OR590584), Puget Sound, Washington (BBPS027-19 in BOLD) and Charleston, Oregon (Fig. 2; Maslakova et al. 2022: OTU 46). The two species closely resemble each other, but one is known to occur from Alaska to Oregon, while the other from Puget Sound, Washington to Long Beach, California. Because the type locality of *L.flavescens* Coe, 1904 is San Pedro, California, we assume that we encountered the “real” *L.flavescens*, and not its more northerly look-alike.

**Habitat.** Collected from among colonial ascidians, algae, and other low intertidal organisms on rocky intertidal shores (stations 2, 6, 10, 15). Also collected just below the surface on intertidal mudflats (station 8).

**Distribution.** Puget Sound, WA, USA (BBPS722-19 in BOLD); Charleston, OR, USA (Hiebert 2016; Maslakova et al. 2022); Bodega Bay (this study), Point Mugu (DISA800-19 in BOLD) and Long Beach, CA, USA (DISA619-19 in BOLD).

**Notes.** With *Kulikovia* sp. BOBA003 (above) and Siphonenteron gen. sp. BOBA004 (below), this species belongs to the Siphonenteron clade, defined by Chernyshev et al. (2018), also referred to as “lineid lineage N” by Kajihara et al. (2022b), and not closely related to the type species of the genus, *Lineuslongissimus* (Gunnerus, 1770).

***Micruraverrilli* Coe, 1901**

*Lineusstriatus* Griffin, 1898: 214.

*Micruraverrilli* Coe, 1901: 68, pl. V, figs 1–3.

**BIN.**BOLD:ADW4746.

**Material examined.** BON2.

**Morphology.** Body ~ 12 cm long, margins and ventral surface white, dorsally with an orange patch at the anterior tip, bordered posteriorly by white, and followed by a broad purple stripe, which is interrupted at intervals by thin, transverse white lines (Fig. 5H). With lateral cephalic slits and a slender caudal cirrus.

**Identification.** Morphologically, specimens from Bodega Bay resemble *Lineusstriatus* briefly described by Griffin (1898) from Puget Sound, Washington and *Micruraverrilli* described by Coe (1901) from Prince William Sound, Alaska. In addition, three similar species have been reported from the western Pacific: *Micrurabella* (Stimpson, 1857), *Micruraimpressa* (Stimpson, 1857), and *Micrurafestiva* Takakura, 1898. Coe synonymized Griffin’s taxon with his own (despite Griffin’s having priority), and *M.impressa* and *M.festiva* and have been treated as synonyms of *M.bella* (Crandall and Norenburg 2001), although *M.impressa* is still listed as an accepted species in WoRMS (WoRMS Editorial Board 2023). ASAP analysis of the COI data suggests that all available *M.verrilli* sequences from the west coast of USA and Canada comprise a single OTU (Fig. 2). A closely related species is reported by Chernyshev and Polyakova (2022) from the Bering Sea as *Evelineus* sp., but there is no mention of its appearance, and it has only been encountered at depths 350 m and below. COI sequences suggest that Bodega Bay specimens are conspecific (97–99% similarity COI) with *M.verrilli* reported from Bamfield, BC, Canada (EF125007), Puget Sound, Washington (KF935508), and Charleston, Oregon (Maslakova et al. 2022: OTU 65). One additional available COI sequence (EF125001) of *M.verrilli* from Puget Sound, Washington contains many ambiguities, which causes some algorithms (e.g., Geneious Prime distance calculations) to interpret it as substantially different from the others, however ASAP analysis places it within the same OTU. A COI sequence of *M.bella* reported from the Sea of Japan (NC_027727) is ~ 10% different from that of *M.verrilli*, and thus belongs to a separate species.

Crandall and Norenburg (2001) suggest the extent of the anterior orange patch may help differentiate the eastern (exclusively dorsal) and western (extends onto ventral side) Pacific forms, however one of the *M.verrilli* specimens found in Washington had pigmentation on both sides (Schwartz 2009).

**Habitat.** Collected from the low intertidal zone among colonial ascidians (station 18) and observed among kelp holdfasts washed ashore in the Bodega Bay region.

**Distribution.** Bamfield, BC, Canada (Schwartz 2009); Puget Sound, WA, USA (Kvist et al. 2014); Charleston, OR, USA (Maslakova et al. 2022); Bodega Bay, CA, USA (this study).

**Notes.** The species reported here belongs to a clade of lineids with orange or magenta red anterior tip that may be synonymous with *Evelineus* (Schwartz 2009), also referred to as “lineid lineage A” (Kajihara et al. 2022b) and is not closely related to the type species of the genus, *Micrurafasciolata* Ehrenberg, 1828 (Chernyshev et al. 2018; Chernyshev and Polyakova 2019).

***Micrurawilsoni* (Coe, 1904)**

*Lineuswilsoni* Coe, 1904: 195, pl. XVI, figs 10, 11.

**BIN.**BOLD:ADW9830.

**Material examined.** BON41.

**Morphology.** Dark brown worm, ~ 45 mm long, slightly paler ventrally, cephalic lobe bordered by white at the anterior tip and along the lateral cephalic slits; thin white transverse bands at irregular intervals along most of the body length (Fig. 5G). No ocelli; rosy cerebral ganglia visible through the body wall. With a small white caudal cirrus.

**Identification.** Conforms to the description of *Micrurawilsoni* (Coe, 1904), described from Monterey and San Pedro, California. No look-alikes are currently known in the northeast Pacific. Although we were not able to obtain a high–quality COI sequence, 16S rRNA sequence from the Bodega Bay individual is 99–100% identical to those of *M.wilsoni* reported by Hiebert (2016) from southern Oregon. These individuals correspond to the COI–delimited *M.wilsoni* of Maslakova et al. (2022: OTU 90).

**Habitat.** Collected from kelp holdfasts (*Macrocystispyrifera*) in the subtidal (station 6) and very low intertidal zones (station 7), also among holdfasts of subtidal bull kelp (*Nereocystisluetkeana*) washed ashore. In southern Oregon found on the exposed rocky shore under boulders and in rock crevices of the low intertidal zone.

**Distribution.** British Columbia, Canada (Gustav Paulay et al., unpublished BOLD records), Charleston, OR, USA (Hiebert 2016; Maslakova et al. 2022); Bodega Bay, CA, USA (this study). Records from San Juan Islands, WA (Maslakova, unpublished) and south to Mexico (Roe et al. 2007) are not currently substantiated by DNA data.

**Notes**. According to a recent phylogenetic analysis of the family Lineidae this species is not closely related to the type species of the genus, *Micrurafasciolata*, but is a member of a clade called “lineid lineage G” by Kajihara et al. (2022b).

**Siphonenteron gen. sp. BOBA004**

**BIN.**BOLD:ADR9817.

**Material examined.** BON59.

**Morphology.** Body 97 mm long, uniformly orange (Fig. 5I). Head the same width as the body, with pale margins, lateral cephalic slits. Ocelli arranged in two rows, one along each anterolateral margin (Fig. 5J), ~ 7 ocelli each, but it is difficult to know the true number as the pigment granules appear broken up and irregular.

**Identification.** BON59 resembles other species from the Siphonenteron clade (defined by Chernyshev et al. 2018), such as *Lineusflavescens*, *Kulikovia* spp. (Kajihara et al. 2022b; this study), and several undescribed representatives of the Siphonenteron clade from southern Oregon previously reported as *Lineus* sp. 1, *Lineus* sp. 2, *Lineus* sp. crescent, *Lineus* sp. red (Hiebert and Maslakova 2015b; Hiebert 2016) or *Lineus* sp. and *Kulikovia* sp. (Maslakova et al. 2022: OTUs 47, 49–51, 59). Among our collections from Bodega Bay, it most resembles *Lineusflavescens* and *Kulikovia* sp. BOBA003 (above). COI and 16S sequences do not have any species-level matches in GenBank; both place this species within the Siphonenteron clade (Figs 2, 3).

**Habitat.** Collected from a wave-exposed, rocky intertidal habitat among colonial ascidians and coralline algae (station 15).

**Distribution.** Discovery Island, Canada (QHAK2948-23 in BOLD); Bodega Bay, CA, USA (this study).

**Notes.** Species new to science.

### ﻿Class HOPLONEMERTEA Hubrecht, 1879

**Order Monostilifera Brinkmann, 1917**

**Suborder Cratenemertea**

**Familial classification suspended as per Kajihara, 2021**

**Genus *Nipponnemertes* Friedrich, 1968**

Of the seven *Nipponnemertes* species described from the northeast Pacific, *N.bimaculata* (Coe, 1901), *N.drepanophoroides* (Griffin, 1898), *N.fernaldi* Iwata, 2001, *N.occidentalis* (Coe, 1905), *N.pacifica* (Coe, 1905), *N.punctatula* (Coe, 1905), and *N.rubella* (Coe, 1905), only two have been reported and/or barcoded in recent years, *N.bimaculata* and *N.punctatula*. The type locality of the latter species is southern California, but the only available barcodes are from Japan (Thollesson and Norenburg 2003; Hookabe et al. 2022), so the two may represent distinct species. An unknown species was reported from southern California (Andrade et al. 2012), from 360–390 m, but its identity and relationship to the above species is not yet known. Clearly, there is a diversity of *Nipponnemertes* species along the Pacific coast of North America, including several cryptic species.

Hookabe et al. (2022), in their revision of the genus that includes descriptions of ten new species, reported three clades of *Nipponnemertes* that are supported by molecular data as well as morphology: degree of head demarcation and presence/absence of a cephalic patch. The species we encountered at Bodega Bay most closely resembles *N.bimaculata* (Coe, 1901), which belongs to Clade B of Hookabe et al. (2022), with the northwest Pacific species *N.jambio*Hookabe et al., 2022, *N.neonilae*Hookabe et al., 2022, *N.ojimaorum*Hookabe et al., 2022, *N.crypta*Hookabe et al., 2022, and a potential trans-Pacific complex of species currently known as *N.punctatula* (Coe, 1905). Species in this complex vary in the degree of development of dorsal and cephalic pigmentation, but most have more or less distinct cephalic pigment patch(es).

***Nipponnemertes* sp. BOBA028**

**BIN.**BOLD:AEJ7531.

**Material examined.** BON13, BON95.

**Figure 6.**
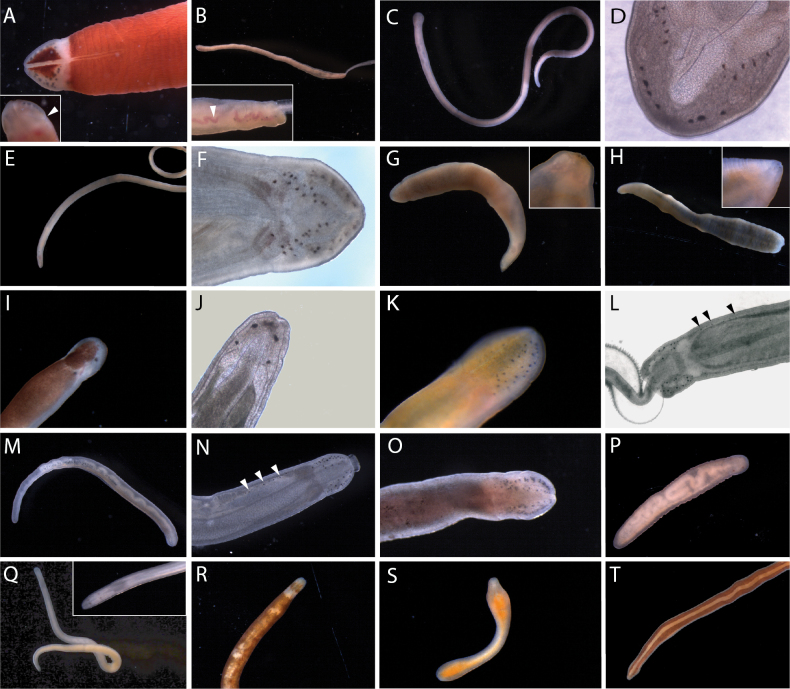
Hoplonemerteans of Bodega Bay **A***Nipponnemertes* sp. BOBA028, a new species, individual BON95. Dorsal and ventral (inset) view of the anterior, showing cerebral organ furrows with numerous secondary furrows (arrowhead) and pink cerebral ganglia **B***Amphiporus* sp. BOBA024, a new species, individual BON61. Inset a close up lateral view of the anterior, showing red blood vessels (arrowhead) **C, D***Amphiporus* sp. BOBA018 individual BON44 **D** close up of head in transmitted light, showing pattern of ocelli **E, F***Amphiporus* sp. BOBA017 individual B20 **F** close up of head in transmitted light, showing pattern of ocelli **G***Poseidonemertes* sp. BOBA010, a new species, individual B14. Inset a close up of the head showing ocelli **H***Poseidonemertes* sp. BOBA033, a new species, individual BON35, full body. Inset a close up of the head showing ocelli **I***Tetrastemmanigrifrons*, anterior end of BON01 **J***Tetrastemma* sp. BOBA029, close up of head of B04 in transmitted light **K***Zygonemertes* sp. BOBA012, individual B09 **L***Zygonemertes* sp. BOBA014, a new species, close up of anterior of BON88 in reflected light, showing post-cerebral ocelli (arrowheads) **M, N***Zygonemertes* sp. BOBA013, individual BON87 **N** close up of anterior showing post cerebral ocelli (arrowheads) **O***Zygonemertes* sp. BOBA015, a new species, individual BON63 **P***Antarctonemertesphyllospadicola*, individual BON67 **Q***Nemertellina* sp. BOBA011, new to science, individual BON69 **R**–**S***Oerstedia* sp. BOBA022, new to science, showing differences in color pattern **R** individual BON32 **S** individual BON33 **T***Tetrastemmabilineatum*, individual B18.

**Morphology.** Body 55–67 mm long, broad, reddish brown dorsally, much paler (almost white) ventrally. Head white, pointed, narrower than the body, with two maroon, triangular pigment patches placed symmetrically on either side of a mid-dorsal ridge (Fig. 6A). Numerous large ocelli lie in the space between the anterolateral margins and the pigment patches, ~ 20 per side. With two pairs of cephalic furrows. The anterior, cerebral organ furrows are equipped with numerous secondary furrows, as is characteristic of other members of the genus (Fig. 6A, inset). The posterior neck furrow forms a posteriorly directed V on the dorsal surface. Pink cerebral ganglia are discernible from the ventral side, just posterior to the cerebral organ furrows (Fig. 6A, inset). Proboscis and stylet apparatus not observed.

**Identification.** The specimens from Bodega Bay conform to the description of *Nipponnemertesbimaculata* (Coe, 1901) except for the shape of the cephalic patches, which are triangular in our specimens as opposed to oval in the original description. However, Coe (1905) later reported triangular patches in other specimens. COI sequences from Bodega Bay individuals form a separate OTU from those of the species previously reported as *N.bimaculata* from Washington and Oregon (Thollesson and Norenburg 2003; Maslakova et al. 2022: OTU 28), as well as an additional species collected in Puget Sound, Washington by CIE (BHFL_5974, Fig. 2), which can be distinguished from other northeast Pacific look-alikes by mottling on the dorsum. The *N.bimaculata* reported from Washington and Oregon seems to have narrower cephalic patches than the species from Bodega Bay. We have not observed any individuals with oval cephalic patches. The type locality of *N.bimaculata* is Alaska and Puget Sound, Washington (Coe 1901), although the species was later reported south to Ensenada, Mexico (Coe 1940). We suggest to reserve the name *N.bimaculata* for the northern form with solid dorsal pigmentation (Maslakova et al. 2022: OTU 28), and to treat Bodega Bay specimens as a new species.

**Habitat.** Collected from the holdfasts of kelp (*Macrocystispyrifera*) in the very low intertidal zone (station 7).

**Distribution.** Bodega Bay, CA, USA (this study).

**Notes.** Species new to science.

### ﻿Suborder EUMONOSTILIFERA Kajihara, 2021

#### ﻿Infraorder AMPHIPORINA Kajihara, 2021

Familial classification suspended as per Kajihara (2021)

**Genus *Amphiporus* Ehrenberg, 1831**

A diverse and non-monophyletic genus of the class Hoplonemertea with 74 species listed in the WoRMS database, many more having been declared *nomen dubium*, or transferred to other genera (Gibson and Crandall 1989). We refer to the species below as *Amphiporus* merely to emphasize the close relationship to previously described species within the genus, not to imply that they constitute a monophyletic group.

***Amphiporus* sp. BOBA024**

**BIN.**BOLD:AEI5687.

**Material examined.** BON36, BON61.

**Figure 7.**
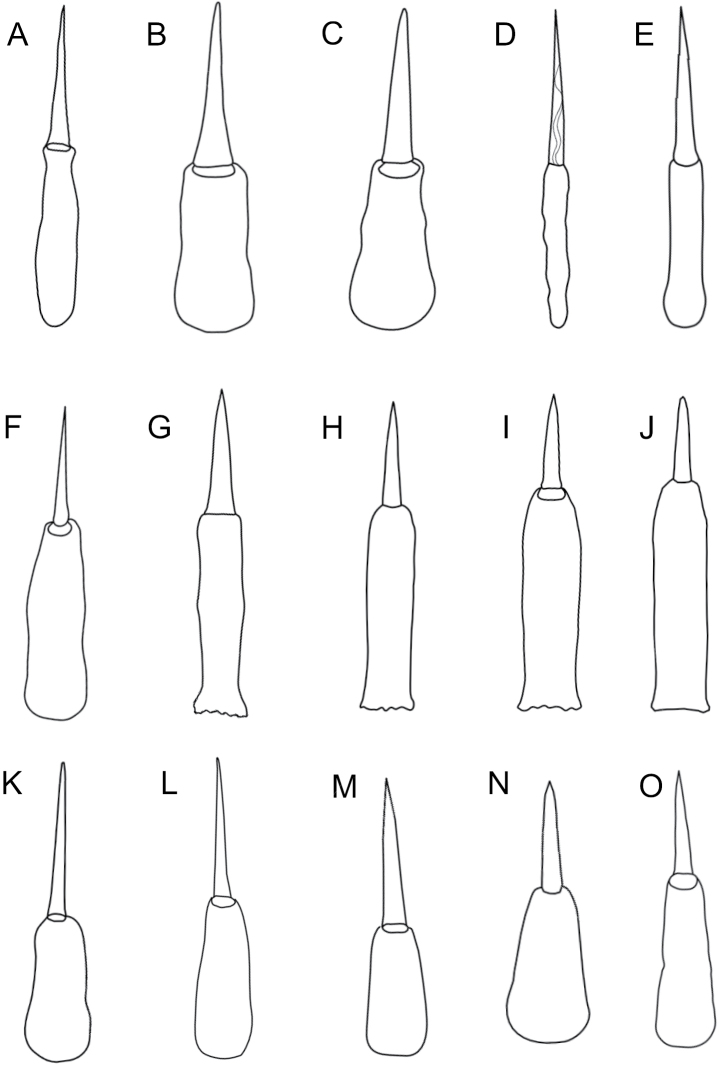
Stylets of Hoplonemerteans of Bodega Bay **A***Amphiporus* sp. BOBA024, individual BON61 **B***Amphiporus* sp. BOBA018, individual BON16 **C***Amphiporus* sp. BOBA017, individual B20 **D***Ototyphlonemertes* sp. BOBA030, individual BON78 **E***Poseidonemertes* sp. BOBA010, individual B14 **F***Tetrastemmanigrifrons*, individual BON01 **G***Zygonemertes* sp. BOBA012, individual B06 **H***Zygonemertes* sp. BOBA014, individual B07 **I***Zygonemertes* sp. BOBA013, individual BON76 **J***Zygonemertes* sp. BOBA015, individual BON62 **K***Antarctonemertesphyllospadicola*, individual BON67 **L***Nemertellina* sp. BOBA011, individual BON69 **M***Oerstedia* sp. BOBA022, individual BON32 **N**Eumonostilifera sp. BOBA016, individual B15 **O***Tetrastemma* sp. BOBA020, individual BON75.

**Morphology.** Body slender, 13 mm long, yellowish white (Fig. 6B). Red blood vessels show prominently through the body wall (Fig. 6B, inset). Head narrow, with a single row of ~ 8 ocelli on either side (Fig. 6B, inset). Very slender, cylindrical basis slightly longer than the central stylet (Fig. 7A), with two accessory stylet pouches.

**Identification.** Specimens from Bodega Bay resemble *Amphiporuscruentatus* Verrill, 1879, originally described from Vineyard Sound, Massachusetts, but later reported from Puget Sound, Washington to San Diego, California (Coe 1905, 1940), in having a small and slender pale yellow body, a single row of ocelli on each side of head, red blood, and a very slender basis of central stylet, with ratio of stylet length to basis length (S/B ratio) close to 1. DNA sequence data are not available for *A.cruentatus* from the Atlantic Coast of North America. Maslakova et al. (2022) published COI sequences of two other Pacific *A.cruentatus* look-alikes: one from southern Oregon (OTU 27), and another from the Bay of Panama (OTU 143), and a third from the Caribbean coast of Panama (OTU 274, as Monostilifera gen. sp.). Given this abundance of cryptic species, it seems likely that the Pacific forms are distinct from the originally described Atlantic *A.cruentatus*. Bodega Bay individuals form a separate OTU from the Oregon individuals (Fig. 2; Maslakova et al. 2022: OTU 27).

**Habitat.** Collected from wave-exposed rocky shores among surfgrass roots and other low intertidal organisms (stations 14, 18).

**Distribution.** Bodega Bay, CA, USA (this study).

**Notes.** Species new to science.

***Amphiporusimparispinosus* Griffin, 1898 species complex**

***Amphiporus* sp. BOBA017**

*Amphiporusimparispinosus*: Maslakova et al. 2022.

**BIN.**BOLD:ADR7530.

**Material examined.** B20, BON47.

**Morphology.** Body 63 mm long, pale yellow to pale peach color (Fig. 6E). Head rounded and wider than the adjacent body, with 20–25 ocelli on each side, arranged as a row along the anterior margin, and another, more irregular grouping, medially (Fig. 6F). Cerebral ganglia pinkish in color. Basis pear-shaped, broadening posteriorly, S/B ~ 1 (Fig. 7C). With three accessory stylet pouches.

**Identification.** Specimens from Bodega Bay conform to the description of *Amphiporusimparispinosus* Griffin, 1898 from Port Townsend, Washington and Sitka, Alaska. Two similar species have been described from the northeast Pacific: *Amphiporusleuciodus* Coe, 1901, from Victoria, BC, Canada and New Metlakatla and Glacier Bay, Alaska, and *Amphiporussimilis* Coe, 1905, from Monterey, California, though Coe later treated the former as a synonym (1905), and the latter as a variety (1940) of *A.imparispinosus*. Subsequent authors retained all three as valid species (Gibson and Crandall 1989; Crandall and Norenburg 2001; WoRMS; but see Roe et al. 2007). Coe (1905) notes that *A.imparispinosus* has a pink brain, small cerebral sense organs, three accessory stylet pouches, and is longer (to 75 mm) than *A.similis*, which is 10–15 mm, with a clear brain, large cerebral sense organs, fewer ocelli and two accessory stylet pouches. Griffin (1898) does not mention color of the cerebral ganglia in *A.imparispinosus*.

Maslakova et al. (2022) report, based on COI sequence data, three distinct *A.imparispinosus*-like species (OTUs 4–6) from the northeast Pacific. One of those (OTU 4) is distributed from Dutch Harbor, AK to Charleston, OR, and overlaps the original range of *A.imparispinosus* (including samples from Puget Sound, WA), so may represent the true *A.imparispinosus*. The other two OTUs have not been reported north of Oregon (Maslakova et al. 2022: OTUs 5 and 6). Another look-alike is reported from the Sea of Japan (Chernyshev and Polyakova 2019: MN211508). The pinkish color of cerebral ganglia and the three accessory stylet pouches in our specimens suggests that it is not *A.similis*, but an undescribed cryptic species.

**Habitat.** Collected from wave-exposed, rocky intertidal habitats (stations 16, 18), including on holdfasts of the kelp *Egregiamenziesii* and within mid-intertidal mussel beds; among algal turf.

**Distribution.** Charleston, OR, USA (Maslakova et al. 2022: OTU 6); Bodega Bay (this study); Point Mugu, CA, USA (DISA798-19 in BOLD).

***Amphiporus* sp. BOBA018**

*Amphiporusimparispinosus*: Maslakova et al. 2022.

**BIN.**BOLD:AEA1922.

**Material examined.** BON16, BON44.

**Morphology.** Body 38–70 mm long, white. Head rounded and wider than body (Fig. 6C). Four clusters of ocelli; two rows following the anterolateral margins, and two more located posteriorly and medially, above the colorless cerebral ganglia (Fig. 6D). The posterior clusters of ocelli appear reddish, while the anterior rows appear brown in reflected light. The neck furrow is obvious and forms a dorsal V-shape posterior to the cerebral ganglia. Basis with rounded posterior margin and slight medial constriction. Central stylet equal in length to the basis (Fig. 7B). Proboscis with three accessory stylet pouches.

**Identification.** See *Amphiporus* sp. BOBA018 above. The presence of three pouches of accessory stylets and the length of the worms suggest that this is not *A.similis*, but an undescribed cryptic species.

**Habitat.** Collected from wave-exposed, rocky intertidal habitats (stations 18, 19), including on holdfasts of the kelp *Egregiamenziesii* and crawling across other low intertidal surfaces.

**Distribution.** Charleston, OR, USA (Maslakova et al. 2022: OTU 5); Bodega Bay, CA, USA (this study).

**Notes.** This is the first record of the species in California.

**Genus *Emplectonema* Stimpson, 1857**

***Emplectonemaviride* Stimpson, 1857**

*Emplectonemaviride* Stimpson, 1857: 163.

*Emplectonemagracile*: Coe 1901: 23, pl. VIII, fig. 3; 1905: 207, pl. I, figs 14, 15; 1940: 279, pl. XXX, fig. 40; Roe et al. 2007: 229, pl. 89, fig. I.

**BIN.**BOLD:AAP1200.

**Material examined.** B16.

**Morphology.** Body long and slender, green dorsally, cream-colored ventrally. Head not especially demarcated from the body. With numerous ocelli distributed along the colorless anterolateral margins of the head. Pink cerebral ganglia. Basis of central stylet much longer than the slightly curved central stylet.

**Identification.** Specimens from Bodega Bay conform to the description of *Emplectonemaviride* Stimpson, 1857, as redescribed by Mendes et al. (2021). There are no known look-alikes in the northeast Pacific. For years, the species has been reported as a synonym of its Atlantic look-alike *Emplectonemagracile* (Johnston, 1837) (e.g., Roe et al. 2007 and references therein). The two species were recently shown to be distinct, the name *E.viride* resurrected, and the Pacific species redescribed (Mendes et al. 2021).

**Habitat.** Collected from a mid-intertidal mussel bed (station 1), and commonly observed in many intertidal habitats throughout the Bodega Bay region. Typically associated with acorn barnacles, which it preys upon.

**Distribution**: Amaknak (MZ580909) and Fox (MZ580901) Islands, AK, USA; Bamfield (MG423290) and Discovery Islands (QHAK2422-22, QHAK2449-22 in BOLD), BC, Canada; Charleston, OR, USA (Hiebert 2016; Mendes et al. 2021; Maslakova et al. 2022; von Dassow et al. 2022); Bodega Bay, CA, USA (this study). The species is reported as far south as Mexico (Roe et al. 2007), but so far, there are no DNA sequence data to confirm.

**Notes.** For photographs of this species, see Mendes et al. (2021: fig. 3)

**Genus *Ototyphlonemertes* Diesing, 1863**

Species of *Ototyphlonemertes* are exclusively mesopsammic, living in the interstices of well-sorted, coarse marine sediments. They are easily distinguished from other small, slender, white eumonostiliferans by the presence of a pair of statocysts in the cerebral ganglia, and the lack of ocelli in adults, but most of the described species appear to represent cryptic species complexes (e.g., Leasi and Norenburg 2014; Leasi et al. 2016). Two species of *Ototyphlonemertes* with spirally sculpted stylets are described from the Pacific coast of the U.S.: *O.americana* Gerner, 1969 and *O.spiralis* Coe, 1940, and a third species, with a smooth stylet, was reported near San Francisco (Roe et al. 2007). However, the *Ototyphlonemertes* spp. of the Pacific coast are not well sampled, and “forms that key out to either of the known species have a reasonable probability of not being those species” (Roe et al. 2007).

***Ototyphlonemertes* sp. BOBA030**

*Ototyphlonemerteslactea*: Leasi et al. 2016.

**BIN.**BOLD:ADM3126.

**Material examined.** BON77, BON78.

**Morphology.** Body 3.5 mm long and less than 1 mm wide, white; foregut region transparent, intestinal region cream-colored. Head slightly demarcated from the body by a transverse cephalic groove. With a pair of statocysts, one in each of the two ventral cerebral ganglia. Statocysts of the polygranular type. Basis slender, irregularly cylindrical, and longer than the spirally sculpted central stylet (Fig. 7D). Proboscis diaphragm not especially long, middle chamber bulbous.

**Identification.** Specimens from Bodega Bay conform to the description of *O.americana* Gerner, 1969 from Puget Sound, Washington, but DNA sequence data are not available from Puget Sound region to confirm identification. COI sequences from Bodega Bay specimens in this study match closely (> 99% similarity, COI) with a species previously reported from San Diego, California as *Ototyphlonemeteslactea* by Leasi et al. (2016: KU230123). *O.lactea* was described from Brazil (Corrêa 1954), but the name was later used to refer to a group of species with similar morphologies and presumed shared ancestry, called phylomorphs (Envall and Norenburg 2001). *O.americana* belongs to the lactea type, which was later synonymized with the macintoshi type (Kajihara et al. 2018), a decision supported by a multigene phylogenetic analysis (Leasi et al. 2016). Relative to macintoshi type worms, *O.americana* and *O.lactea* have a shorter proboscis diaphragm and a bulbous middle chamber, as opposed to a long, tubular one. Another OTU of a lactea type reported from Half Moon Bay, California by Leasi et al. (2016: KU230128) is likely to be confused with the Bodega Bay species. Sampling of *Ototyphylonemertes* in Puget Sound is needed to resolve the specific identity of these lineages.

**Habitat.** Collected from low intertidal, coarse marine sediments on a wave-exposed sandy beach (station 13).

**Distribution.** Wright’s Beach, CA, USA (Leasi et al. 2016); Bodega Bay, CA, USA (this study).

**Genus *Paranemertes* Coe, 1901**

***Paranemertes* sp. BOBA009**

*Paranemertesperegrina*: Hiebert 2016: 78.

*Paranemertes* sp.: Hao et al. 2015: 572, fig. 1J.

**BIN.**BOLD:ADM0221.

**Material examined.** B2.

**Morphology.** Body ~ 5 cm long, orangish purple dorsally, paler ventrally. Head slightly demarcated from the body, with red cerebral ganglia visible through the body wall. Stylet apparatus not observed.

**Identification.***Paranemertesperegrina* is a cryptic species complex composed of at least seven distinct lineages (Hao et al. 2015). A single widely distributed lineage likely corresponding to the *P.peregrina* Coe, 1901 originally described from Alaska has been identified on the basis of proximity to the type locality and habitat, and is confirmed by DNA sequence data to occur from British Columbia, Canada to southern Oregon, USA on the northeast Pacific coast, and from Kuril Islands, Russia to Shandong, China on the northwest Pacific coast (Hao et al. 2015). The remaining six species, including the two previously reported from Bodega Bay, remain undescribed.

**Habitat.** Collected from kelp holdfasts in shallow subtidal zone within Bodega Harbor (station 6).

**Distribution.** Unalaska Island, AK, USA (DUTCH345-19 in BOLD); Discovery Islands, BC, Canada (QHAK177-20 in BOLD); Charleston, OR, USA (Hiebert 2016; Maslakova et al. 2022: OTU 10); Bodega Bay, CA, USA (Hao et al. 2015; this study).

**Notes.**Hao et al. (2015) report that the two Paranemertescf.peregrinaOTUs from Bodega Bay are well-separated by habitat, with one occurring in mudflats and the other in rocky intertidal contexts, under stones, among algae, etc. Interestingly, the species encountered in this survey came from among kelp holdfasts but matches the one previously found in mudflats by Hao et al. (2015). This might not be as surprising as it appears at first because the kelp holdfasts collected by us (station 6) were from a small kelp bed within Bodega Harbor immediately adjacent to extensive mudflats. For photographs of this species, see Hao et al. (2015: fig. 1J).

**Genus *Poseidonemertes* Kirsteuer, 1967**

***Poseidonemertes* sp. BOBA010**

**BIN.**BOLD:AEK1697.

**Material examined.** B14.

**Morphology.** Stout, pale, rust-colored worm with thick, clear margins; body widens posteriorly. Anterior end sharply pointed, with two ocelli near its tip (Fig. 6G). Basis cylindrical, slender, of a similar length as the central stylet (Fig. 7E). With two accessory stylet pouches.

**Identification.** The two *Poseidonemertes* specimens from this study (B14 and BON35 listed below) resemble *Poseidonemertescollaris* Roe & Wickham, 1984 described from Bodega Bay, California, and other light-colored members of the genus, e.g., *Poseidonemertesmaslakovae* Chernyshev, 2002 and *Poseidonemertes* sp. 508 from the Sea of Japan. *P.collaris* is the only member of the genus previously reported from the Pacific coast of the U.S. COI sequence data suggest B14 represents a distinct OTU (8.2% divergent) from what was reported as *P.collaris* by Thollesson and Norenburg (2003) and from BON35 (described below, 19% divergent), or any other previously sequenced members of the genus.

**Habitat.** Collected just offshore, < 200 m from an open coast beach, among subtidal sand/mud sediments from a depth of 6–7 m (station 3).

**Distribution.** Bodega Bay, CA, USA (this study).

**Notes.** Species new to science.

***Poseidonemertes* sp. BOBA033**

**BIN.**BOLD:AEK1698.

**Material examined.** BON35.

**Morphology.** Stout, cream-colored worm, ~ 25 mm long, with branched intestinal diverticula, greenish in color, highly visible through the body wall (Fig. 6H). Body widens posteriorly. Head pointed, with two ocelli near its tip (Fig. 6H, inset). Stylet apparatus not observed.

**Identification.** See B14 above.

**Habitat.** Collected from an intertidal mudflat, just below the surface of the sediment, among polychaete tubes (station 8).

**Distribution.** Bodega Bay, CA, USA (this study).

**Notes.** Species new to science.

**Genus *Tetrastemma* Ehrenberg, 1831**

This non-monophyletic genus of small four eyed eumonostiliferans containing > 100 species was recently redefined by Chernyshev et al. (2021a) based on a multigene phylogenetic analysis. *Tetrastemmanigrifrons* is part of the *Tetrastemma* clade sensu Chernyshev et al. 2021a. The other two species are included here tentatively.

***Tetrastemmanigrifrons* Coe, 1904**

*Tetrastemmanigrifrons* Coe, 1904: 159, pl. XV, fig. 7, pl. XVI, figs 6–9, pl. XVII, fig. 1, pl. XX, fig. 16, pl. XXI, figs 15–23; Maslakova et al. 2022.

*Quasitetrastemmanigrifrons* Chernyshev, 2004: 154; Chernyshev et al. 2021a.

**BIN.**BOLD:ADX0572.

**Material examined.** BON1.

**Morphology.** Body 27 mm long, brown dorsally, pale ventrally. Head rounded with colorless margins and a broad brown patch, differentiated only slightly from the body by the colorless transverse band (Fig. 6I). With four eyes; the anterior pair halfway between the anterior tip and the cerebral organ furrows, the posterior pair just below the posterior furrow. With bright red blood vessels. Cylindrical basis, slightly longer than the central stylet (Fig. 7F). With two accessory stylet pouches.

**Identification.** Fits the description of *Tetrastemmanigrifrons* Coe, 1904, described from Monterey Bay, California. A look-alike, *Tetrastemmastimpsoni* Chernyshev, 1992 occurs in the northwest Pacific and the Sea of Japan (Chernyshev et al. 2021a). A third, closely related species has been documented from Dutch Harbor, Alaska (6–7% divergence, BIN: BOLD:AEC4254). No pictures are available, but the description (“brown dorsally, white band separates head, 2 prs eye spots”) matches that of this species.

**Habitat.** Collected from the low intertidal zone among red algal blades and colonies of the kamptozoan *Barentsiaconferta* (station 17). Similar specimens also observed intertidally among low zone tunicates and algae (station 2), kelp holdfasts (station 7), and surfgrass roots (station 14). Subtidally, among organisms on marina docks (station 9).

**Distribution.** Unalaska, AK, USA (DUTCH209-19 in BOLD); Canada (Chernyshev et al. 2021a); Charleston, OR, USA (Chernyshev et al. 2021a; Maslakova et al. 2022); CA, USA (Chernyshev et al. 2021a); Bodega Bay, CA, USA (this study).

**Notes.** This species has variable coloration, both in terms of pattern and the amount of pigmentation (Roe et al. 2007; Maslakova, unpublished). Some specimens are almost completely dark brown dorsally with a colorless transverse bar separating the cephalic patch from the dorsum, while others have much less dorsal pigment (e.g., it may be separated into two more or less continuous stripes by a pigment-less mid-dorsal region). Some specimens may be almost entirely devoid of pigment, except for the cephalic patch (which may be broken into two by a mid-dorsal gap). With four ocelli. Blood vessels are red, and clearly show through the body wall.

***Tetrastemma* sp. BOBA029**

*Tetrastemma* sp. 1: Hiebert 2016: 84, fig. 2.14.

*Tetrastemma* sp.: Maslakova et al. 2022.

**BIN.**BOLD:ADW8618.

**Material examined.** B4.

**Morphology.** Small and slender, transparent, fast-moving worm; internal structures appear yellowish through the body wall. Anterior tip with a small white patch. With four ocelli (Fig. 6J). Stylet apparatus not observed.

**Identification.** Anterior white patch and otherwise featureless body distinguishes this from other species of *Tetrastemma* reported from northeast Pacific (Roe et al. 2007). DNA sequences from the Bodega Bay specimen match those of *Tetrastemma* sp. 1 first reported from southern Oregon by Hiebert (2016) and Maslakova et al. (2022: OTU 19 as *Tetrastemma* sp.). Two other overall similar species lacking the anterior white patch occur in southern Oregon (Maslakova et al. 2022: OTUs 15 and 20).

**Habitat.** Collected from Bodega Harbor within the holdfasts of subtidal Giant Kelp (*Macrocystispyrifera*) at a depth of 3–4 m (station 6). In Oregon, collected from among surfgrass (*Phyllospadix* spp.) in the rocky intertidal zone (Hiebert 2016).

**Distribution.** Charleston, OR, USA (Maslakova et al. 2022); Bodega Bay, CA, USA (this study).

**Notes.** First record of the species in California. This species is very common in southern Oregon. Reproductive individuals were found in July in southern Oregon, and deposited egg masses in laboratory dishes upon collection, with crawl-away juveniles hatching a week or two later.

***Tetrastemma* sp. BOBA020**

**BIN.**BOLD:AEJ7493.

**Material examined.** BON75.

**Morphology.** Body 1.7 mm long, transparent, with an orange gut. With four eyes and two pairs of cephalic furrows: cerebral organ furrows at the level of the posterior pair of eyes, and a V-shaped neck furrow posteriorly, overlying the anterior portion of the cerebral ganglia. Conical basis, significantly longer than the central stylet (Fig. 7O).

**Identification.** Resembles other featureless species of *Tetrastemma.* COI sequences show it to be distinct from any previously sequenced species.

**Habitat.** Collected from low intertidal, coarse marine sediments on a wave-exposed sandy beach (station 13).

**Distribution.** Bodega Bay, CA, USA (this study).

**Notes.** Species new to science.

**Genus *Zygonemertes* Montgomery, 1897**

Members of the genus *Zygonemertes* are distinct from other eumonostiliferans in having a single row of post cerebral ocelli on each side, along the lateral nerve cords, in addition to the more typical ocelli found in rows or groups on the head. In addition, all species we have had the opportunity to examine possess sickle-shaped microscopic inclusions in the epidermis, and most have a characteristically truncated basis of the central stylet.

Three species of *Zygonemertes* are reported from the northeast Pacific coast: *Z.albida* Coe, 1901, *Z.thalassina* Coe, 1901, and *Z.virescens* (Verrill, 1879). The first two were described by Coe from British Columbia and Alaska, respectively. *Zygonemertesthallasina* has never been reported outside its type locality, *Z.albida* was subsequently reported by Coe to occur as far south as Ensenada, Mexico (Coe 1944), and *Z.virescens* is reported to have a very wide geographic distribution including Pacific (British Columbia to Mexico), Atlantic (Maine to Florida) and Gulf coasts of North America, as well as Curaçao (Coe 1940; Gibson 1995; Roe et al. 2007), but clearly represents a large cryptic species complex (Maslakova, unpublished). The type locality of *Z.virescens* is New England. Presently we are aware of several genetically distinct Atlantic look-alikes (e.g., from Florida, Colombia, and Caribbean Panama); these are also distinct from several Pacific *Z.virescens*-like forms. Based on this, it seems most reasonable to exclude *Z.virescens* from the list of Pacific fauna, and to describe the Pacific forms as new species.

*Zygonemertesthalassina* was regarded as being extremely similar to *Z.virescens*, except often longer (to 60 mm), darker in color (olive green), with a smaller S/B ratio, a shorter, stubbier central stylet, and with five stylets per accessory pouch, rather than two or three (Coe 1901, 1905). *Zygonemertesalbida* was distinguished on the basis of its small size, lack of color, longer proboscis, and differences in the stylet apparatus, appearing similar to juveniles of the other two species. Among individuals of *Z.virescens*, Coe noted variation in color, number and arrangement of ocelli, and relative proportions of central stylet and basis. While some of these features may be variable (e.g., with age or environment), we consider it likely that he encountered more than one species, as there are at least four *Z.virescens*-like species in southern Oregon alone (Maslakova et al. 2022: OTUs 23–26), and we identified an additional two species in this study. The increasing number of *Zygonemertes* species uncovered with genetic data, and the lack of barcodes from type localities make it difficult to assign existing names to these species. For now, we refer to them as *Zygonemertes* spp. until formal descriptions are made. We consider reports of *Z.albida* from the Atlantic coast dubious (Zattara et al. 2019).

***Zygonemertes* sp. BOBA012**

*Zygonemertes* sp. 1: Hiebert 2016: 70.

*Zygonemertes* sp.: Maslakova et al. 2022.

Nemertea sp.: Leray and Paulay unpublished (MH242861).

**BIN.**BOLD:ADL9636.

**Material examined.** B5, B6, B9.

**Morphology.** Body somewhat transparent, greenish yellow with clear margins; 15–25 ocelli arranged in two irregular rows on each side of the head, with a single row of post cerebral ocelli on each side along the lateral nerve cords. Cerebral ganglia pink, visible through the body wall (Fig. 6K). With two pairs of cephalic furrows; cerebral organ furrows are simple ventrolateral arches located 1/2 to 2/3 of the way between the anterior tip and the neck furrow, which overlays the anterior portion of the cerebral ganglia. Specimens B5 and B9 had long slender basis with a slightly concave to flat posterior margin, B6 had a flared stylet basis similar to the illustration for *Z.virescens* in Roe et al. (2007). Central stylet shorter than basis, S/B ~ 0.5 (Fig. 7G); two accessory stylet pouches, with two stylets each.

**Identification.** See above on species of *Zygonemertes*. COI sequences from Bodega Bay specimens match those of Nemertea sp. from Puget Sound, Washington (Paulay and Leray, unpublished, MH242862) and those reported as *Zygonemertes* sp. 1 (Hiebert 2016) or *Zygonemertes* sp. (Maslakova et al. 2022: OTU 23) from southern Oregon.

**Habitat.** Collected from Bodega Harbor within the holdfasts of subtidal Giant Kelp (*Macrocystispyrifera*) at a depth of 3–4 m (station 6).

**Distribution.** Puget Sound, WA, USA (MH242861; Maslakova, unpublished); Charleston, OR, USA (Maslakova et al. 2022); Bodega Bay, CA, USA (this study).

**Notes.** Species not previously reported from California. In the first round of PCR with universal primers, we apparently amplified the gut contents of these worms, the barnacle *Balanusglandula*. Like another barnacle-eating nemertean, *Emplectonemaviride*, some specimens of this species have a long, slender basis of central stylet. The basis in this species appears slightly narrower than in other species of *Zygonemertes*.

***Zygonemertes* sp. BOBA013**

*Zygonemertes* sp. 1: Hiebert 2016: 70.

*Zygonemertes* sp.: Maslakova et al. 2022; O’Mahoney et al. unpublished (MZ580839).

**BIN.**BOLD:ADW7912.

**Material examined.** BON27, BON76, BON80, BON87.

**Morphology.** Body 4–15 mm long; color ranging from white with a tinge of yellow to orange, sometimes with dark pigment spots along the sides of the body or at the posterior (Fig. 6M). 25–50+ ocelli arranged in four irregular rows on the head, a single row of ~ 10 post-cerebral ocelli along the lateral nerve cords (Fig. 6N). With two pairs of cephalic furrows: cerebral organ furrows are simple ventrolateral arches located 2/3 of the distance from the tip of the head to the V-shaped neck furrow, which overlies the cerebral ganglia. Basis slender, much longer than the central stylet, S/B ~ 0.5–0.65, sometimes with slight medial constriction posteriorly, and with flat or slightly concave posterior margin (Fig. 7I). Two accessory stylet pouches with two or three stylets each.

**Identification.** See above on species of *Zygonemertes*. COI sequences from Bodega Bay specimens match a subset of those reported as *Zygonemertes* sp. 1 (Hiebert 2016) and *Zygonemertes* sp. (Maslakova et al. 2022: OTU 26) from southern Oregon, and two specimens from Dutch Harbor, Alaska. This species may correspond to *Z.albida* or a cryptic undescribed species.

**Habitat.** Collected from the low intertidal zone among colonial ascidians and polychaete worm tubes (stations 7, 10). Collected subtidally from within holdfasts of bull kelp (*Nereocystisluetkeana*), station 11.

**Distribution.** Amaknak (MZ580839) and Unalaska Islands, AK, USA (MZ580813); San Juan Island, WA, USA (Maslakova, unpublished), Charleston, OR, USA (Hiebert 2016; Maslakova et al. 2022); Bodega Bay, CA, USA (this study).

**Notes.** First record of the species in California. Reproductive individuals encountered in August in Bodega Bay.

***Zygonemertes* sp. BOBA014**

**BIN.**BOLD:AEK0256.

**Material examined.** B7, B8, BON88, BON91.

**Morphology.** Body up to 31 mm long, greenish yellow, digestive tract appearing bright reddish orange ventrally. With ~ 15 ocelli on each side of the head, arranged in four irregular rows, and a single row of post-cerebral ocelli (~ 8) along each lateral nerve cord (Fig. 6L). Cerebral organ furrows 2/3 the distance between the anterior tip and the posterior V-shaped neck furrow. Cylindrical basis, longer than the central stylet, with truncated posterior margin (Fig. 7H). S/B ~ 0.5–0.65. Two accessory stylet pouches, with two or three stylets each.

**Identification.** See above on species of *Zygonemertes*.

**Habitat.** Collected from Bodega Harbor within the holdfasts of subtidal Giant Kelp (*Macrocystispyrifera*) in the shallow subtidal zone (< 5 m depth, station 6).

**Distribution.** Bodega Bay, CA, USA (this study).

**Notes.** Species new to science. Closely related to *Zygonemertes* sp. BOBA015 (6% divergence, COI).

***Zygonemertes* sp. BOBA015**

**BIN.**BOLD:AEJ0120, BOLD:ADR7155.

**Material examined.** BON62, BON63, BON81.

**Morphology.** Body 4–15 mm long, brownish, with numerous ocelli arranged in four irregular rows on the head, ~ 25+ on each side (Fig. 6O). Basis quite massive compared to central stylet, in terms of length and width (Fig. 7J), though this is not as obvious in smaller specimens (BON63). S/B 0.4–0.75. One individual (BON81) had an unusual triangle-shaped basis, widening significantly posteriorly, with a flat posterior margin. Two accessory stylet pouches with two or three stylets each.

**Identification.** See above on species of *Zygonemertes*.

**Habitat.** Low intertidal zone among surfgrass roots (station 14) and colonial ascidians (station 10).

**Distribution.** Calvert Island, BC, Canada (BHAK2541-20 in BOLD). Bodega Bay, CA, USA (this study); Point Mugu, CA, USA (DISA797-19 in BOLD).

**Notes.** Species new to science. Closely related to *Zygonemertes* sp. BOBA014 (6% divergence, COI).

**Amphiporina incertae sedis**

**Eumonostilifera sp. BOBA016**

Monostilifera sp.: Maslakova et al. 2022.

**BIN.**BOLD:AEJ6897.

**Material examined.** B15.

**Morphology.** Body orange, with 15 ocelli on each side of the cephalic lobe. Basis nearly conical, rounded at the bottom, a bit shorter than the central stylet (Fig. 7N). Two accessory stylet pouches with two stylets each.

**Identification.** Resembles individuals of *Amphiporus* sp. BOBA017 and BOBA018 described above, but COI sequences do not match any previously sequenced species, and do not group closely with *Amphiporus* cf. imparispinosus. Taxonomic affiliation is uncertain until a more thorough phylogenetic analysis (with more conservative markers than COI and 16S) is carried out. Overall morphology and 16S tree (Fig. 3) suggest it belongs within Amphiporina.

**Habitat.** Among fouling organisms on marina docks (station 9).

**Distribution.** San Juan Island, WA, USA (Maslakova, unpublished); Charleston, OR, USA (Maslakova et al. 2022); Bodega Bay, CA, USA (this study).

**Notes.** First record of the species in California. The eggs of this species (which have a polyhedral chorion) have been collected in the plankton in Charleston, OR (Maslakova et al. 2022: OTU 13) and matching COI barcodes have been obtained from adults collected from Friday Harbor, WA (Maslakova, unpublished).

**Infraorder Oerstediina Kajihara, 2021**

**Family Oerstediidae Chernyshev, 1993**

**Genus *Antarctonemertes* Friedrich, 1955**

***Antarctonemertesphyllospadicola* (Stricker, 1985)**

*Tetrastemmaphyllospadicola* Stricker, 1985: 682, figs 1–28; Stricker and Cavey 1986: 2188; McDermott 1997: 254; Stricker and Folsom 1997: 57; Stricker et al. 2001: 214.

**BIN.**BOLD:ACH3602.

**Material examined.** BON67, BON68.

**Morphology.** Body short and stout, pale yellow to pale peach color, 6–7 mm long (Fig. 6P), with four eyes occupying the corners of a square, and a prominent pointed snout (not apparent on Fig. 6P). Cephalic lobe at its widest at the level of cerebral organ furrow, between the first and second pairs of eyes. Cerebral organ furrows are limited to the ventral side. Transverse neck furrow posterior to the second pair of eyes. Rounded in cross section, proboscis extending to posterior end of the body. Basis oval, widening a bit posteriorly, with a slender central stylet, S > B (Fig. 7K). With two accessory stylet pouches, with one or two stylets each.

**Identification.** Specimens from Bodega Bay conform to the description of *Antarctonemertesphyllospadicola* (Stricker, 1985) described from San Juan Island, Washington, and the COI sequences match those of *A.phyllospadicola* from San Juan Island, Washington (Thollesson and Norenburg 2003) and southern Oregon (Maslakova, unpublished).

**Habitat.** Collected with intertidal samples of surfgrass, *Phyllospadixscouleri* (station 14). In the San Juan Islands, WA this species is found on blades and inside female inflorescences of *P.scouleri* in the low intertidal zone.

**Distribution.** Bamfield Marine Science Centre, Canada (Chernyshev and Polyakova 2019); Puget Sound, WA, USA (Maslakova and von Döhren 2009); Charleston, OR (Maslakova, unpublished); Bodega Bay, CA, USA (this study).

**Notes.** This is the first record of this genus and species for California.

**Genus *Nemertellina* Friedrich, 1935**

The genus *Nemertellina* has never been reported from the northeast Pacific and currently contains five valid species, three occurring in Kiel Bay, Germany, and one each in Madagascar and Japan. Members of this genus have four eyes, with the anterior and posterior pairs widely separated; small and simple cerebral organs located far anterior to the brain and opening ventrally near the tip of the head; short rhynchocoel; conical or pear-shaped basis, with 2–4 accessory stylet pouches. *Nemertellinacanea* Friedrich, 1935b, *N.minuta* Friedrich, 1935a, *N.oculata* Friedrich, 1935b and *N.tropica* Kirsteuer, 1965 are reported to completely lack cephalic furrows, while *N.yamaokai* Kajihara, Gibson & Mawatari, 2000 has two pairs.

***Nemertellina* sp. BOBA011**

**BIN.**BOLD:AEJ4336.

**Material examined.** B3, B21, BON69.

**Morphology.** Body small and slender, ~ 15 mm long, cylindrical in cross-section (Fig. 6Q). Head the same width as adjacent body. Four eyes occupy the corners of a rectangle, the distance between the anterior and posterior pairs of eyes is considerably larger than the distance between the two eyes of each pair. With two pairs of cephalic furrows: cerebral organ furrows are just posterior to the anterior pair of eyes, and the posterior neck furrow overlies the anterior margin of the cerebral ganglia, which are translucent. Stylet basis cylindrical, rounded posteriorly, S/B ~ 1 (Fig. 7L); two accessory stylet pouches. The rhynchocoel extends 3/4 of the body length.

**Identification.** The species encountered here is most similar to *Nemertellinayamaokai* in possessing two sets of cephalic furrows. The Bodega Bay specimens are ~ 10% divergent (COI) from *N.yamaokai*, suggesting the presence of a sixth *Nemertellina* species, and the first reported from the northeast Pacific.

**Habitat.** Collected subtidally in Bodega Harbor from kelp holdfasts (station 6) and among fouling organisms on boat marina docks (station 9). Collected intertidally from rocky shores on the open coast among surfgrass roots (station 14).

**Distribution.** Charleston, OR, USA (Maslakova, unpublished); Bodega Bay, CA, USA (this study).

**Notes.** Species new to science, and new record of the genus for North America.

**Genus *Oerstedia* Quatrefages, 1846**

***Oerstedia* sp. BOBA022**

**BIN.**BOLD:AEJ2779.

**Material examined.** BON32, BON33.

**Morphology.** Short and stout cylindrical body, 3–6 mm long, with a head narrower than the body. One individual pale, with a bright orange gut (Fig. 6S), the other with the dorsal surface completely covered with blotches of various shades of brown (Fig. 6R). With four large eyes. Basis conical, rounded posteriorly, shorter than the slender central stylet (Fig. 7M). Two accessory stylet pouches, with three or four stylets each.

**Identification.** The only species of *Oerstedia* reported to occur in the northeast Pacific (from Washington to Mexico) is *Oerstediadorsalis* (Abildgaard, 1806). The type locality of *O.dorsalis* is northern Europe, but the species has been reported throughout the northern hemisphere and is famously polymorphic. Sundberg et al. (2009) demonstrated that there are at least nine cryptic species within *O.dorsalis* in northern Europe alone, each exhibiting color polymorphism. Reports of *Oerstedia* on this coast likely refer to undescribed species. Bodega Bay specimens are sufficiently divergent from any previously sequenced *Oerstedia*, including a species occurring in southern Oregon (Maslakova, unpublished).

**Habitat.** Collected among low intertidal red algae on the rocky boulders of a breakwater (station 4).

**Distribution.** Bodega Bay, CA, USA (this study).

**Notes.** Species new to science.

**Oerstediina incertae sedis**

***Tetrastemmabilineatum* Coe, 1904**

*Tetrastemmabilineatum* Coe, 1904: 164, pl. XIV, fig. 6, pl. XXI, figs 13, 14, pl. XXII, fig. 4.

**BIN.**BOLD:ADW8130.

**Material examined.** B18.

**Morphology.** Small, slender worm with two dorsal longitudinal brown stripes, each 1/3 of the body width, upon a cream-colored background (Fig. 6T). Brown stripes narrow and terminate towards the anterior tip. Head triangular in shape, with four eyes; the anterior pair located halfway between the tip of the head and the posterior pair.

**Identification.** Specimens from Bodega Bay conform to the description of *Tetrastemmabilineatum* Coe, 1904, originally from San Diego, California, though sequence data are not available from southern California. COI sequence of the Bodega Bay specimen matches those of *T.bilineatum* individuals reported from southern Oregon (Hiebert 2016; Maslakova et al. 2022: OTU 29).

**Habitat.** Collected from a wave-exposed mussel bed (station 1) and found in similar habitat in southern Oregon.

**Distribution.** Bamfield Marine Science Centre, Canada (Chernyshev et al. 2021a); Charleston, OR, USA (Hiebert 2016; Maslakova et al. 2022); Bodega Bay, CA, USA (this study).

**Notes.**Coe’s (1904) original record of the species from San Diego, California is not verified by DNA sequence data, but given the distinctiveness of this species, and the absence of known look-alikes on this coast, the reported distribution seems likely. According to a recent molecular phylogeny of *Tetrastemma* and its allies (Chernyshev et al. 2021a) this species does not belong to *Tetrastemma* sensu stricto or the infraorder Amphiporina, but instead is a member of Oerstediina. Its generic placement remains uncertain.

## ﻿Discussion

The geographic distributions and abundances of coastal species are changing in response to a variety of human impacts (O’Hara et al. 2021), including warming oceans (Sagarin et al. 1999; Sorte et al. 2010; Lonhart et al. 2019; Sanford et al. 2019). Evaluation of these ecological changes is hindered by our incomplete knowledge of the fauna and flora of coastal ecosystems, pointing to a critical need for assessment of biodiversity (Gray 1997). Currently, up to 90% of marine eukaryotic species are estimated to remain undescribed (Mora et al. 2011; Appeltans et al. 2012). Lesser-studied groups, such as nemerteans, have an especially high fraction of undescribed and undetected species (e.g., Hiebert 2016; Maslakova et al. 2022).

Our study extends the geographic focus of recent taxonomic work on nemerteans to include northern California where relatively little work has been done on nemertean diversity during the past 60 years. Notably, only 13 of the 34 species (38%) we collected and barcoded can be unambiguously assigned to described species. This highlights that nemertean diversity remains poorly known in the northeast Pacific despite more than a century of study. That the majority of the observed diversity cannot be assigned to described species renders the few existing geographically relevant identification guides (e.g., Coe 1940; Corrêa 1964; Kozloff 1996; Roe et al. 2007) inadequate.

Eleven species (32%) reported here are new to science, and ten (29%) comprise previously reported undescribed species, or cryptic species whose taxonomic status cannot be resolved with data at hand (Table 4). Some of the newly discovered species were likely overlooked or set aside because of their relatively small size and evident cryptic morphology (e.g., *Tetrastemma* sp. BOBA020, *Nemertellina* sp. BOBA011). Others represent cryptic lineages of previously described species (e.g., *Amphiporuscruentatus*, *Amphiporusimparispinosus*, *Lineusflavescens*, *Nipponnemertesbimaculata*, *Oerstediadorsalis*, *Poseidonemertescollaris*, *Zygonemertesvirescens*). Yet others may have escaped notice due to their rarity or cryptic habits.

Twenty two of the 34 species have not been previously confirmed by DNA barcodes to occur in northern California. This includes two species that appear to have been introduced from other parts of the world (*Cephalothrixsimula* from the northwest Pacific, and *Cephalothrixhermaphroditica* from European waters, possibly via Chile or another point of entry along the Pacific Coast of the Americas). In fact, our study is the first to report *C.hermaphroditica* from the northeast Pacific. Introduction of *C.simula* may be of concern to aquaculture due to its association with oysters, and high levels of tetrodotoxin in its tissues (Kajihara et al. 2013; Turner et al. 2018). These are the first reports in California of two previously described species, *Antarctonemertesphyllospadicola* and *Maculauraoregonensis*, and six previously reported but undescribed species (*Kulikovia* sp. BOBA003, *Amphiporus* sp. BOBA018, *Tetrastemma* sp. BOBA029, *Zygonemertes* sp. BOBA012, 13, *Eumonostilifera* sp. BOBA016). This is also the first record of the genus *Riserius* in California and *Nemertellina* in the northeast Pacific.

Historical surveys of nemertean diversity in the Bodega Bay region (Tamura 1957; Corrêa 1964; Standing et al. 1975; Ristau et al. 1978) identified 21 different species. The majority of these species were also found in our surveys, although in some cases, taxonomic uncertainty makes a direct comparison difficult.

Several species recorded in historical surveys of the Bodega Bay region were absent from our surveys. For example, Corrêa (1964) reported a single specimen of *Lineuspictifrons*, a species we did not locate. Corrêa (1964) also reported several species (e.g., *Tubulanuscingulatus*, *Cerebratuluslongiceps*) that were dredged with soft sediments at 6 m depth in Tomales Bay, a subtidal habitat that we did not survey. Perhaps the most conspicuous absence in our survey was the lack of *Tubulanusruber* (Griffin, 1898), which was reported under the name *Tubulanuspolymorphus* in three of the historical studies cited above and was described as “rather common” in the Bodega Bay region by Corrêa (1964). DNA barcodes suggest that *T.ruber*, originally described from Alaska, is distinct from *Tubulanuspolymorphus* Renier, 1804, an Atlantic species with which it was previously synonymized by Coe (1940) (Hiebert 2016). The geographic range of *T.ruber* (as *T.polymorphus*) has been reported as San Luis Obispo, CA to Alaska (Morris et al. 1980), and from Monterey, CA to the Aleutian Islands (Roe et al. 2007). iNaturalist documents only two clear records of *Tubulanus “polymorphus*” from central California (San Mateo County, iNaturalist 19743045; and San Luis Obispo County, iNaturalist 84371695), and many records from northern California (Mendocino County) to Alaska. While seasonality could potentially explain the absence of some previously documented species in our surveys, one of us (ES) has conducted intertidal fieldwork in this region throughout the year for the past 20 years and has never observed *T.ruber* in Sonoma County. The scarcity of recent observations of this large and conspicuous red/orange ribbon worm from Bodega Bay and the southern portion of its geographic range is consistent with a geographic range contraction.

## ﻿Conclusions

Our findings demonstrate how much there is to learn about the diversity and distribution of nemerteans of the northeast Pacific, particularly among southern regions that have received the least amount of attention. Lack of baseline occurrence data (supported by DNA barcodes) hinders our ability to detect shifts in the distribution and abundance of these species. Further sampling and DNA barcoding along the west coast of North America is needed to obtain a more accurate picture of the diversity in this region. Sampling type localities of previously described species will help resolve some of the taxonomic ambiguities associated with species already encountered.

## ﻿Additional information

### Author ORCIDs

Christina I. Ellison https://orcid.org/0000-0002-1856-386X

Eric Sanford https://orcid.org/0000-0001-9053-6826

Svetlana Maslakova https://orcid.org/0000-0002-3629-6638

### Data availability

All of the data that support the findings of this study are available in the main text or Supplementary Information.

## Supplementary materials

Supplementary material 1 Reference sequences used in species delimitation (ASAP), alignments, and trees Data type xlsx https://binary.pensoft.net/file/1062155This dataset is made available under the Open Database License (http://opendatacommons.org/licenses/odbl/1.0/). The Open Database License (ODbL) is a license agreement intended to allow users to freely share, modify, and use this Dataset while maintaining this same freedom for others, provided that the original source and author(s) are credited.Christina I. Ellison, Madeline R. Frey, Eric Sanford, Svetlana Maslakova
